# A new oxime synthesized from *Senecio nutans* SCh. Bip (chachacoma) reduces calcium influx in the vascular contractile response in rat aorta†

**DOI:** 10.1039/d4ra01058b

**Published:** 2024-03-25

**Authors:** Javier Palacios, Daniel Asunción-Alvarez, Diego Aravena, Mario Chiong, Marcelo A. Catalán, Claudio Parra, Fredi Cifuentes, Adrián Paredes

**Affiliations:** a Laboratorio de Bioquímica Aplicada, Facultad de Ciencias de la Salud, Universidad Arturo Prat Iquique 1110939 Chile clpalaci@unap.cl holbertasuncion.pharm@gmail.com diego.andresx21@gmail.com; b Universidad de Chile, Advanced Center for Chronic Diseases (ACCDiS), Facultad de Ciencias Químicas y Farmacéuticas Santiago Chile mchiong@ciq.uchile.cl; c Instituto de Fisiología, Facultad de Medicina, Universidad Austral de Chile Valdivia 5090000 Chile marcelo.catalan@uach.cl; d Departamento de Química Orgánica, Facultad de Ciencias Químicas, Universidad de Concepción Edmundo Larenas 129 Concepción 4070371 Chile cparra@udec.cl; e Laboratorio de Fisiología Experimental (EPhyL), Instituto Antofagasta (IA), Universidad de Antofagasta Antofagasta 1271155 Chile fredi.cifuentes.jorquera@gmail.com; f Departamento de Química, Facultad de Ciencias Básicas, Universidad de Antofagasta Antofagasta 1271155 Chile adrian.paredes@uantof.cl

## Abstract

*Senecio nutans* Sch. Bip is an endemic plant commonly employed in the Andes culture to counteract the effects of mountain sickness, and its bioactive molecules could provide new drugs for treating hypertension. The purpose was to determine whether the vascular response of the plant bioactive molecules, such as (5-acetyl-6-hydroxy-2-isopropenyl-2,3-dihydrobenzofurane; Sn–I), could be improved by a simple structural modification to synthesize oximes (Ox–Sn–I). We characterized both compounds using IR and NMR spectroscopy and Heteronuclear Multiple Quantum Coherence (HMQC). We investigated vascular relaxation mechanisms in response to Sn–I and Ox–Sn–I using rat aorta and vascular smooth muscle cells (A7r5) as experimental models. Preincubation of aortic rings with Sn–I (10^−5^ M) significantly (*p* < 0.001) decreased the contractile effect in response to phenylephrine (PE) and potassium chloride (KCl). The sensitivity (EC_50_) to PE significantly (*p* < 0.01) decreased in the presence of Sn–I (10^−5^ M), but not with Ox–Sn–I. Sn–I significantly (*p* < 0.001) reduced the PE-induced contraction under calcium-free conditions. When A7r5 cells were preincubated with Sn–I and Ox–Sn–I (10^−5^ M), both compounds blunted the increase in intracellular Ca^2+^ induced by KCl. 2,3-Dihydrobenzofurane derived from *S. nutans* (Sn–I) reduces the contractile response probably by blocking Ca^2+^ entry through voltage-gated Ca^2+^ channels (VGCC) in vascular smooth cells. This effect also causes relaxation in rat aorta mediated by reduction of intracellular Ca^2+^ concentration, rather than an increase of NO generation in endothelial vascular cells.

## Abstract 2

The Sn–I metabolite decreases the contractile response to KCl or phenylephrine (PE) mainly by blocking Ca^2+^ entry through voltage-gated Ca^2+^ channel (VGCC).

## Graphical abstract

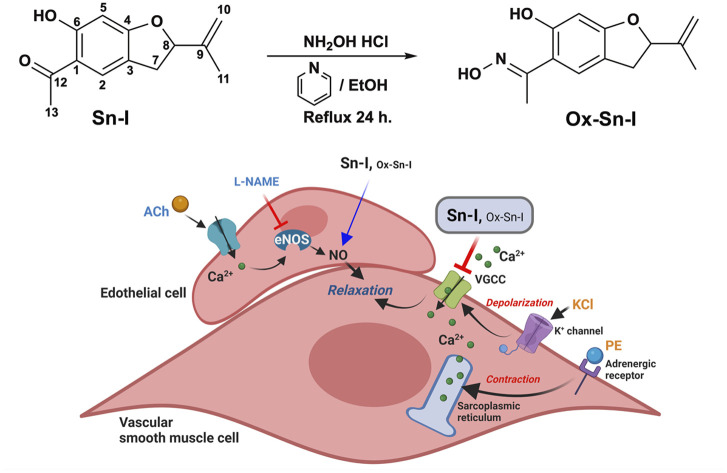

## Introduction

Over the past few decades, village people have sought new alternative medicines, herbal medicines, to cure various diseases, such as hypertension.^1^ Both Andean communities and the village culture of northern Chile often use medicinal plants as a source of pharmaceuticals.^2^ The most representative species of the area used to prevent mountain sickness are *Senecio nutans* Sch. Bip,^3,4^ and *Xenophyllum poposum* (Phil) V.A. Funk,^5^ also referred to as “Chachacoma” and “Popusa”, respectively.^6^

In *S. nutans*, 51 compounds were identified by high-resolution mass spectrometry (UHPLC-MS), such as simple organic acids, amino acids, acetophenones and related compounds, phenolic acids, oxylipins, flavonoids, and coumarins.^7^ In *X. poposum*, 19 compounds were identified by UHPLC-MS, such as isomers caffeoylquinic acid, flavonoids, and acetophenones.^5^ Interestingly, two compounds were isolated in both plants, 4-hydroxy-3-(3-methyl-2-butenyl) acetophenone and 5-acetyl-6-hydroxy-2-isopropenyl-2,3-dihydrobenzofurane.^4,5^

In previous studies, we demonstrated that the extracts of *S. nutans* and *X. poposum* significantly reduced the blood pressure in mice, due to a decrease in atrial sinus rhythm and negative inotropic effect.^3,5^ Moreover, both *S. nutans* and *X. poposum* decrease the contractile response to PE in vascular smooth muscle by reducing the calcium influx from the extracellular space in an endothelium-dependent way.^4,5^

Interestingly, a few metabolites are present in both species, *S. nutans* and *X. poposum*.^4,5^ One of them, 4-hydroxy-3-(isopenten-2-yl)-acetophenone showed a significant endothelium-dependent vasodilatation.^8^

Recently, the synthesis of oxime compounds has become very interesting because they could generate nitric oxide (NO) and cause vasodilation in vascular tissue.^9^ This chemical strategy could be key in the treatment of endothelial vascular dysfunction.^9^ In a previous study, we reported the synthesis of the novel oxime from 4-hydroxy-3-(isopenten-2-yl)-acetophenone and demonstrated that the vasodilator effect was significantly enhanced in the vascular smooth muscle of rat aorta compared to 4-hydroxy-3-(isopenten-2-yl)-acetophenone. Also, we found that the oxime drastically reduced the calcium-dependent contractile response, suggesting that chemical modification of metabolite was successful for vascular response.^8^

Here, we show for the first time the vascular effect of 5-acetyl-6-hydroxy-2-isopropenyl-2,3-dihydrobenzofurane (Sn–I), isolated metabolite from *S. nutans* and its oxime (Ox–Sn–I). Also, this metabolite is also present in *X. poposum*.^5^ This study was designed to enhance the vascular response of the plant bioactive molecules, such as (5-acetyl-6-hydroxy-2-isopropenyl-2,3-dihydrobenzofurane; Sn–I), by a simple structural modification to synthetize of oximes (Ox–Sn–I). Furthermore, better understanding of the vascular relaxation mechanisms of Sn–I and Ox–Sn–I, which could involve the generation of endothelial NO and the influx of extracellular Ca^2+^ in rat aorta.

## Results and discussion

### Separation and structural elucidation of the metabolite Sn–I from *Senecio nutans*

The aerial parts, branches, leaves and flowers of *S. nutans* were extracted as described in the experimental section. The DCM extract was subjected to semi-pressure flash column chromatography packed with silica gel and using a mixture of increasing polarity *n*-hex–EtOAc, obtaining four main subfractions (A–D). Fraction B was subjected to a new column chromatography with silica gel and of increasing polarity of *n*-hex : EtOAc (0–100%) from which a total of 85 fractions were obtained that were grouped into four new subfractions according to the similarity of the chromatographic profile in TLC and in comparison with pure samples of the Sn–I compound. One of these fractions presented a large amount of crystals, which were washed and recrystallized in cold with *n*-hex : EtOAc to obtain the compound of interest.

The Sn–I compound forms colorless crystals, mp. 69–70 °C and molecular weight *m*/*z* 218.1055 assignable to C_13_H_14_O_3_ (calculated 218.0943). The IR spectrum shows a broad band between 3250 and 3500 cm^−1^ that is associated with the presence of an OH group, a series of low intensity absorption bands between 1700 and 1900 cm^−1^ and 1300–1500 cm^−1^ attributable to the presence of an aromatic ring and an absorption band at 1640 cm^−1^ corresponding to an aromatic ketone carbonyl group (see ESI†).

**Fig. 1:**
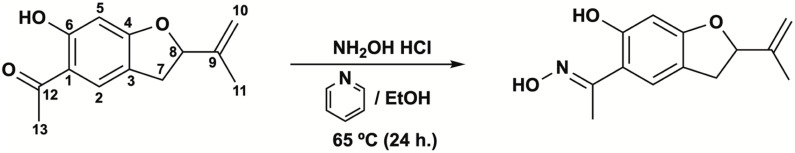
Synthesis the oxime from secondary metabolites isolated from *S. nutans*: 5-acetyl-6-hydroxy-2-isopropenyl-2,3-dihydrobenzofurane (6-hydroxytremetone; dihydroeuparin, left) (Sn–I) and (5-acetyl-6-hydroxy-2-isopropenyl-2,3-dihydrobenzofurane, right) (Ox–Sn–I).

**Fig. 2:**
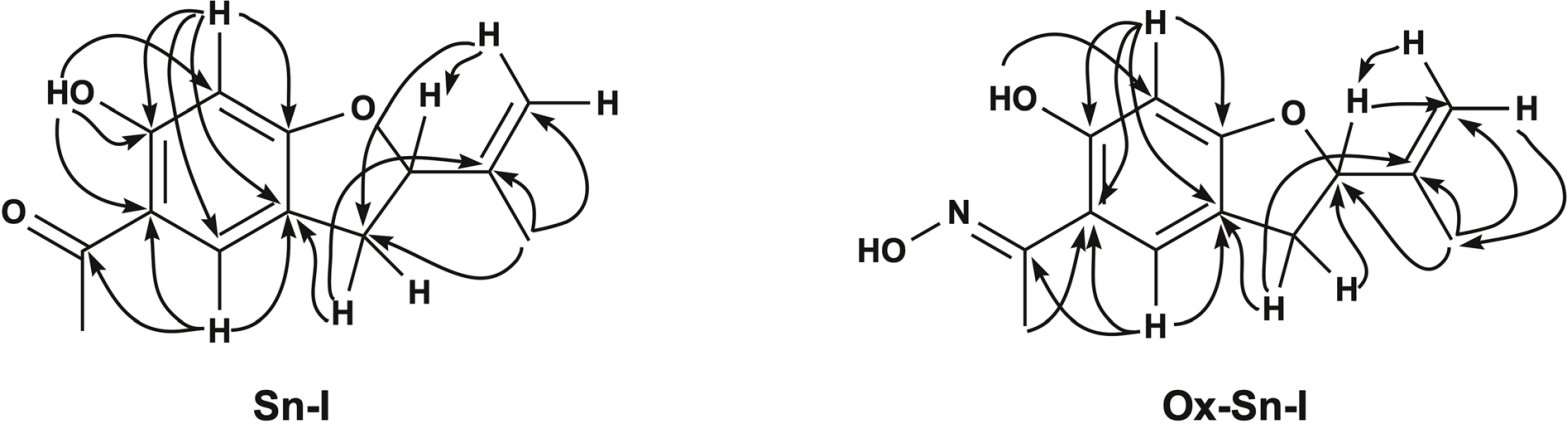
Selected key HMBC (arrows) 2D NMR correlations of compound Sn–I (left) and Ox–Sn–I (right).

The ^1^H-NMR spectrum (Table 1) for Sn–I shows signals assigned to the presence of 14 hydrogen atoms, and the ^13^C-NMR spectrum (Table 1) shows signals for 13 carbon atoms, respectively. The chemical shifts and the DEPT135 spectrum indicate the presence of two CH_3_ groups, one CH_2_ binding to the aromatic ring, one sp^2^-type CH_2_, two aromatic CHs, one sp^3^ quaternary C and six sp^2^-type quaternary carbons. The HMBC spectrum allows establishing the following relationships, the proton that resonates at *δ* 6.36 ppm is linked to the carbon atom at *δ* 98.06 ppm assignable to C-5, the proton at *δ* 7.48 with the carbon at *δ* 126.67 ppm (C-2), the proton at *δ* 5.26 with the carbon at *δ* 87.60 ppm (C-8). While the C sp^2^ that resonates at *δ* 112.77 ppm (C-10) is bound to the protons at *δ* 5.07 and *δ* 4.93 ppm, respectively. The spectroscopic information of the Sn–I compound is the same as described for 6-hydroxy-2-isopropenyl-5-acetyl-2,3-dihydrobenzofuran (dihydroeuparin) (Fig. 1). The coupling patterns deduced from the HMBC spectrum are indicated in Table 1 and Fig. 2.

**Table 1:** Spectroscopic data of compounds Sn–I and Ox–Sn–I in CDCl_3_ (*δ* in ppm, *J* in Hz)^a^

Position	Sn–I			Ox–Sn–I		
	*δ*_H_	*δ*_C_	HMBC	*δ*_H_	*δ*_C_	HMBC
1	—	113.72	—	—	111.39	—
2	7.48 (t) (1.4)	126.67	C-1, C-3, C-12	7.43 (s)	123.32	C-1, C-3, C-12
3	—	118.61	—	—	117.73	—
4	—	166.61	—	—	161.94	—
5	6.36 (s)	98.06	C-2, C-3, C-4, C-6	7.19 (s)	98.23	C-1, C-3, C-4, C-6
6	—	165.76		—	159.47	—
7	2.96 (ddd) (15.4; 7.4; 1.4)	33.11	C-3, C-9	2.97 (dd) (15.1; 7.6)	33.81	C-3, C-8, C-9
	3.00 (ddd) (15.3; 9.5; 1.3)			3.29 (dd) (15.1; 9.6)		
8	5.26 (m) (8.7)	87.60	—	4.91 (d) (8.4)	86.93	C-10
9	—	143.13	—	—	143.71	—
10	5.07 (dt) (1.7; 0.9)	112.77	C-7, C-8	5.21 (t) (8.6)	112.27	C-8, C-11
	4.93 (m) (1.5)			5.07 (s)		
11	1.75 (s)	16.98	C-7, C-9, C-10	1.75 (s)	17.04	C-8, C-9, C-10
12	—	201.98	—	—	159.23	—
13	2.53 (s)	26.23	—	2.31 (s)	10.88	C-1
OH	12.98 (s)	—	C-1, C-5, C-6	11.70	—	C-5

a Spectra recorded at 400 MHz for ^1^H NMR and at 100 MHz for ^13^C NMR.

### Synthesis and purification of Ox–Sn–I from the Sn–I metabolite

Due to the biological activity found in the Sn–I metabolite,^4^ it was decided to carry out a structural modification of the carbonyl group, with a bioisosteric equivalent, an oxime, with the aim of being able to enhance the biological activity with respect to its precursor metabolite. The synthesis of the Sn–I oxime was carried out under the conditions described in the Experimental section. The quantification of the product allowed us to establish that the yield for this synthesis was around 76.56%.

The oxime 6-hydroxy-2-isopropenyl-5-acetyloxime-2,3-dihydrobenzofuran (Ox–Sn–I) (Fig. 1) crystallizes from dichloromethane as green–gray crystals, with mp 76–78 °C and a molecular weight *m*/*z* of 233.1137, which is assignable to C_13_H_15_NO_3_.

The IR spectrum shows low intensity absorption bands between 1800–2000 cm^−1^ and 1400–1500 cm^−1^ assignable to the presence of a benzene ring, a broad absorption band between 3000–3500 cm^−1^ is associated with the presence of vibrations of a –C=N–OH group and a hydroxyl group, and an intense band at 1625 cm^−1^ product of the –C=N–OH interactions of the oximes.

The ^1^H and ^13^C NMR spectra show signals assignable to 14 hydrogen atoms and 13 carbon atoms, respectively. The chemical shifts and the DEPT135 spectrum (Table 1) clearly indicate the presence of two CH_3_ groups, one CH_2_ bonding to the aromatic ring, one sp^2^-type CH_2_, two aromatic CHs, one sp^3^ quaternary C and six sp^2^-type quaternary carbons. One of these carbons corresponds to the ketoxime function (C=N–OH) (C-12), which is evidenced by the shift of the signal to high field, in the ^13^C NMR spectrum this carbon resonates at *δ* 159.23 ppm (C-12), while the carbonyl group of the precursor (C-12) resonates at *δ* 201.98 ppm (Table 1). One of the methyl groups corresponds to a ketoxime group (CH_3_–C=N), this methyl is displaced towards the high field *δ* 2.31 ppm, with respect to the Sn–I compound *δ* 2.53 ppm. This is also evident in the ^13^C NMR spectrum, where the carbon of this group (CH_3_–C=N) resonates at *δ* 10.88 ppm and the precursor resonates at *δ* 26.23 ppm (C-13). The HMBC spectrum allows establishing basically the same relationships, shown for the compound 6-hydroxy-2-isopropenyl-5-acetyl-2,3-dihydrobenzofuran (Sn–I) (Fig. 2).

### Role of the vascular endothelium in the aorta relaxation

**Fig. 3:**
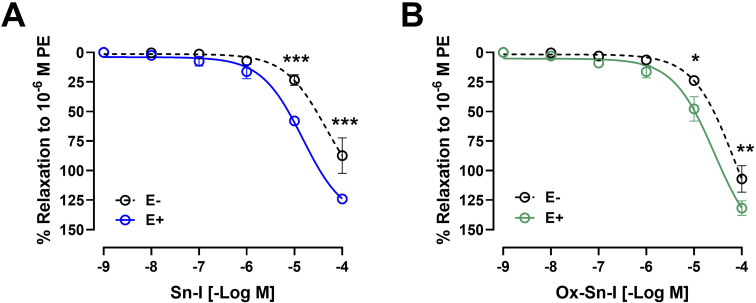
Metabolite Sn–I and its oxime Ox–Sn–I cause endothelium-dependent relaxation effect in rat aorta. Aortic rings were pre-contracted with 10^−6^ M PE, and cumulative concentrations of molecules (10^−9^ to 10^−4^ M) were added in bath. The protocol was repeated in intact rat aorta (E^+^), and endothelium-denuded aorta (E^−^; dashed line) (A and B). Data are the average ± SEM of 4–6 independent experiments. *p < 0.05; ***p < 0.001.

Original recordings showed that both compounds (Sn–I and its oxime Ox–Sn–I) produced vascular relaxation in intact aorta, in a dose-dependent manner (Fig. 3A and 3B). The Sn–I relaxation was significantly (*p* < 0.05) higher than Ox–Sn–I in intact rat aorta: 58 ± 2% Sn–I and 48 ± 10% Ox–Sn–I (10^−5^ M).

The endothelium denudation of aortic rings significantly blunted the relaxation in response to both compounds: Sn–I (58 ± 2% endothelium *vs.* 23 ± 4% denuded-endothelium, 10^−5^ M, *p* < 0.001; Fig. 3A) *versus* oxime Ox–Sn–I (48 ± 10% endothelium *vs.* 24 ± 1% denuded-endothelium, 10^−5^ M, *p* < 0.05, Fig. 3B). This result was confirmed in Table 2: the half maximal inhibitory concentration (IC_50_) of Sn–I was significantly (*p* < 0.01) higher in denuded-endothelium aorta (44.73 ± 6.05 μM) *versus* intact aorta (14.36 ± 8.59 μM), but not with Ox–Sn–I.

**Table 2:** Half maximal inhibitory concentration (IC_50_ μM) to different bioactive molecules. The protocol was repeated in the intact aorta (E^+^), denuded-endothelium aorta (E^−^), or in the presence of l-NAME (10^−4^ M)^a^

Compound	Endothelium (E^+^)	Denuded-endothelium (E^−^)	l-NAME
Sn–I	14.36 ± 8.59	44.73 ± 6.05**	59.42 ± 8.59***
Ox–Sn–I	25.59 ± 7.87	68.14 ± 6.69	89.72 ± 5.38

a The values correspond to mean ± standard error of the mean (SEM) obtained from 4–6 independent experiments. Statistically significant difference **p < 0.01; ***p < 0.001 *vs.* endothelium.

**Fig. 4:**
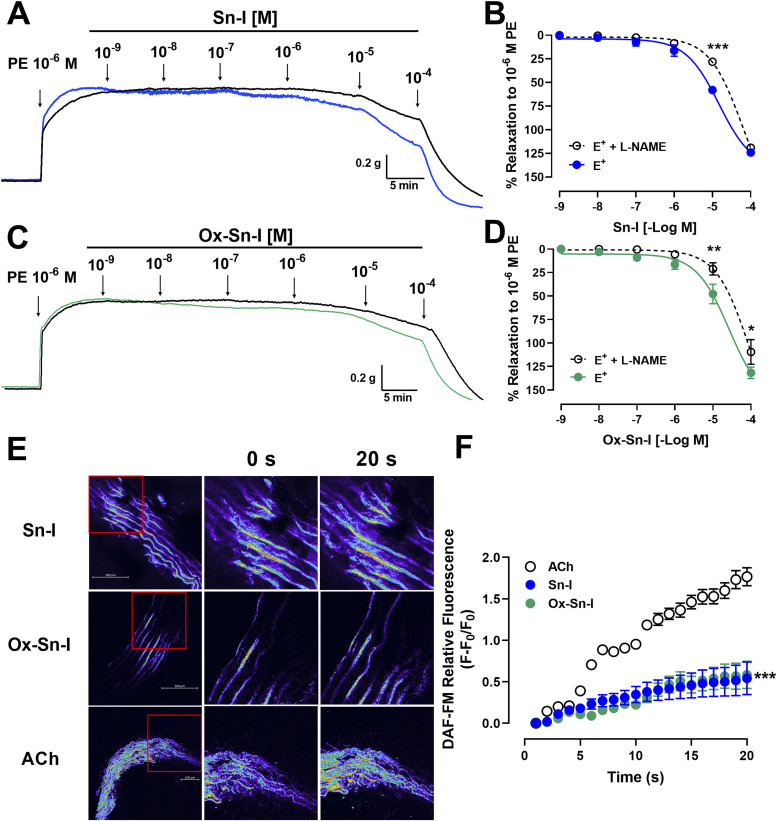
Metabolite and oxime cause relaxation in rat aorta *via* endothelial nitric oxide pathway. Intact aortic rings (E^+^) were pre-incubated with l-NAME (black line; 10^−4^ M) for 20 min. Subsequently, aortic rings were pre-contracted with PE (10^−6^ M), and cumulative doses (10^−9^ to 10^−4^ M) of Sn–I (A and B) and Ox–Sn–I (C and D) were added in bath. Micrograph (20×) of rat aorta sections (E), and fluorescence relative to DAF-FM (nitric oxide) caused by Sn–I, Ox–Sn–I and ACh (10^−5^ M) (F). Data are the average standard error of the mean (SEM) of 4–5 independent experiments. *p < 0.05; **p < 0.01; ***p < 0.001.

To evaluate the role of NO in the response to compounds, aortic rings were treated with l-NAME (an eNOS inhibitor).^10^ Preincubation of intact aortic rings with l-NAME (10^−4^ M) significantly decreased relaxation of both compounds (10^−5^ M): for Sn–I (58 ± 2% *vs.* 28 ± 3% l-NAME; *p* < 0.001; Fig. 4A and 4B), and for Ox–Sn–I (48 ± 10% *vs.* 21 ± 6% l-NAME; *p* < 0.01; Fig. 4C and 4D). The IC_50_ value for Sn–I was significantly (*p* < 0.001) higher in presence of l-NAME (10^−4^ M) than that obtained from the intact endothelium (Table 2).

To evaluate whether Sn–I and Ox–Sn–I release NO from vascular endothelium, experiments were performed on rat aorta slices loaded with DAF-FM (a NO fluorescent probe; 10 μM). Results of relative fluorescence showed that Sn–I and Ox–Sn–I produce a small generation of NO compared with that induced by acetylcholine (10^−5^ M) (Fig. 4E and F).

### Effect of bioactive molecules on the contractile response to PE and KCl

**Fig. 5:**
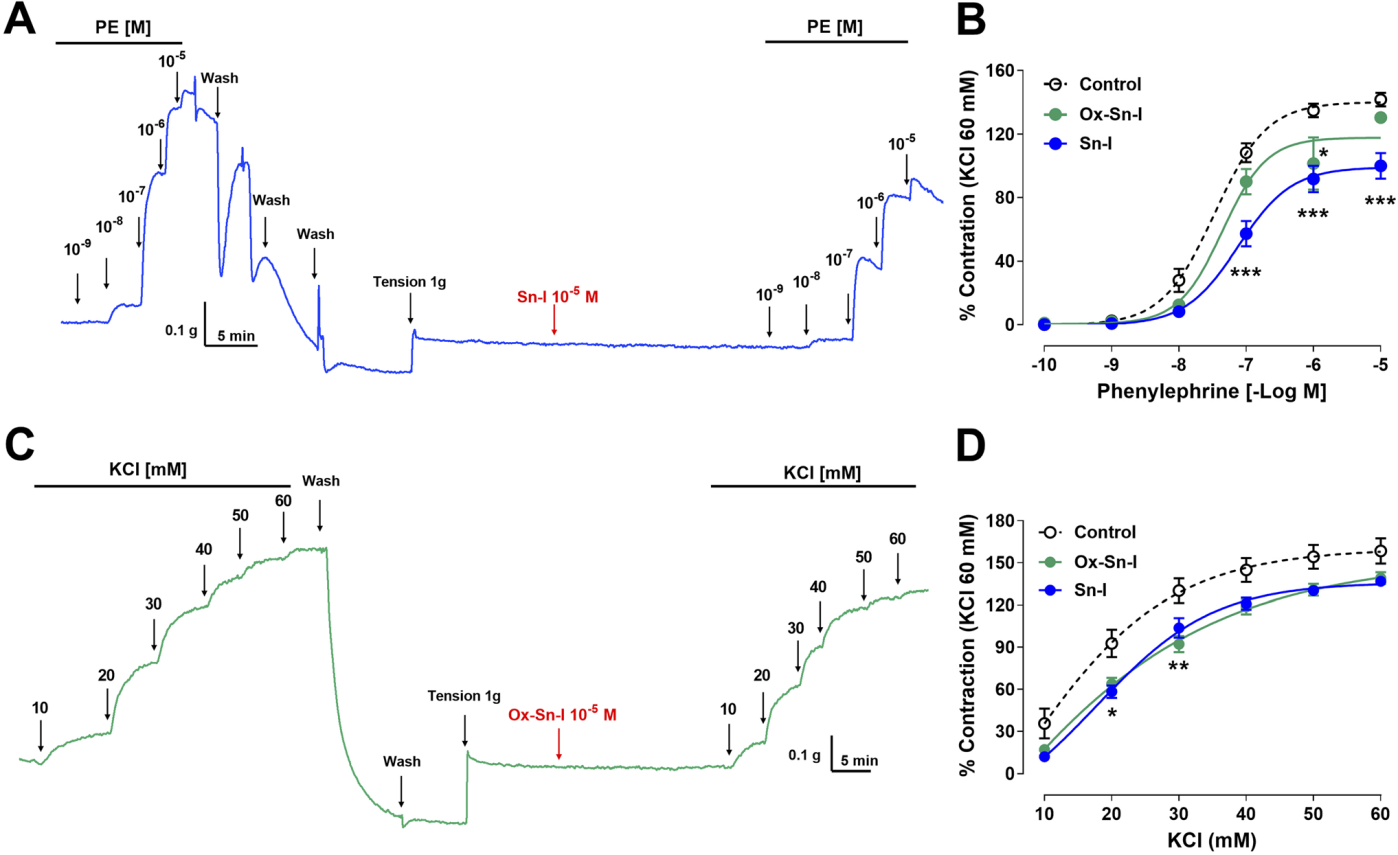
Reduction of the vascular contractile response to PE and KCl by Sn–I and Ox–Sn–I. Intact aortic rings were preincubated with compounds (10^−5^ M) for 20 min. Subsequently, aortic rings were contracted with cumulative concentrations of PE (10^−10^ to 10^−5^ M) (A and B) or KCl (10 to 60 mM) (C and D). Data are the average ± SEM of 4 independent experiments. *p < 0.05; **p < 0.01; ***p < 0.001 *versus* control.

The following experiment was to determine if the relaxation induced by both compounds reduces the contractile response to PE. Preincubation with Sn–I (10^−5^ M) significantly (*p* < 0.001) reduced the vascular contraction of rat aorta in response to PE: 108 ± 6% control *versus* 57 ± 8% Sn–I, 10^−7^ M PE, and similar results were observed to 10^−6^ M and 10^−5^ M PE in rat aorta preincubated with Sn–I (Fig. 5A and B). Conversely, Ox–Sn–I (10^−5^ M) only reduced the contractile response to 10^−6^ M PE (135 ± 4% control *versus* 102 ± 17% Ox–Sn–I, *p* < 0.05, Fig. 5B). The sensitivity (EC_50_) to PE significantly (*p* < 0.01) decreased in the presence of Sn–I (10^−5^ M), but not with Ox–Sn–I (Table 3).

**Table 3:** Half maximal effective concentration (EC_50_ μM) of PE (10^−10^ to 10^−5^ M) or KCl (10 to 60 mM) in intact aortic rings preincubated with bioactive molecules^a^

Compound	PE (nM)	KCl (mM)
Control	34.04 ± 8.49	22.37 ± 1.40
Sn–I	77.06 ± 7.69**	24.21 ± 0.76
Ox–Sn–I	45.72 ± 6.84	26.15 ± 0.93

a The values are mean ± SEM and represent the mean of at 4 independent experiments. Statistically significant difference **p < 0.01 *vs.* control.

The next step was to determine whether the vascular relaxation effect of compounds implicates membrane depolarization by KCl. The results showed that the preincubation of aortic rings with Sn–I (10^−5^ M) significantly (*p* < 0.05) decreased the contractile response to KCl (20 mM) compared to the intact aorta: 93 ± 10% control *vs.* 58 ± 4% Sn–I (Fig. 5C and D). Also, the Ox–Sn–I significantly (*p* < 0.01) reduced the contractile response to KCl (30 mM): 130 ± 9% control *versus* 92 ± 6% Ox–Sn–I (30 mM KCl; Fig. 5D). The sensitivity (EC_50_) to KCl did not vary in the presence of Sn–I, or Ox–Sn–I (Table 2).

### Bioactive molecules reduce the intracellular calcium concentration in the contractile response to PE and KCl

**Fig. 6:**
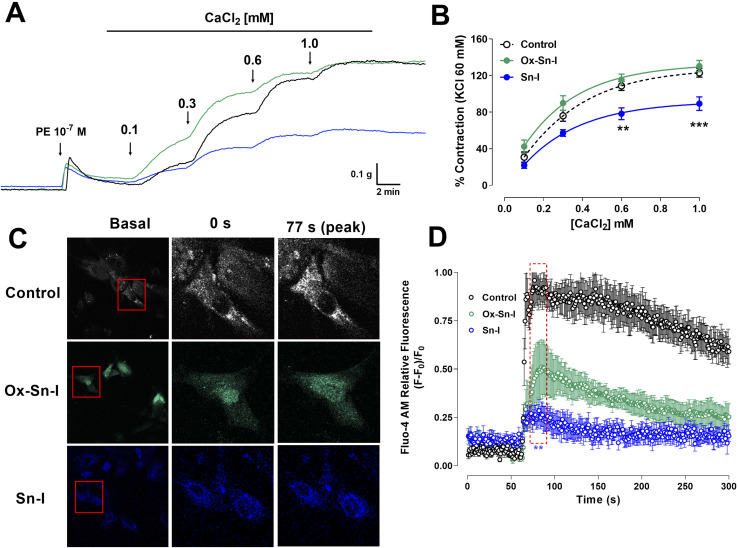
Sn–I and Ox–Sn–I decrease the intracellular calcium in the vascular response to PE and KCl. Original trace and data showing the concentration–response curves to CaCl_2_ in aortic rings precontracted with PE (10^−7^ M) in free-Ca^2+^ medium (A and B); micrograph (10×) of vascular smooth muscle cell line A7r5 (C), and dose–response curves of the effect of compounds on KCl (50 mM) (D). Data are the average ± SEM of 4 independent experiments. **p < 0.01; ***p < 0.001 *versus* control.

We studied the role of extracellular calcium in the contractile response to PE. Firstly, rat aorta was preincubated with Sn–I and Ox–Sn–I (10^−5^ M) in the calcium-free medium, and then PE (10^−7^ M) was added to the bath to induce contractile response by the release of intracellular Ca^2+^ from the sarcoplasmic reticulum. The pre-incubation with Sn–I (10^−5^ M) significantly (*p* < 0.05) decreased the adrenergic-induced contraction in a free-calcium medium compared to control (36 ± 2% control; 29 ± 1% Sn–I; data not shown). Afterward, a cumulative concentration of extracellular calcium (0.1 to 1.0 mM) was added to the medium. Under these conditions, Sn–I significantly (*p* < 0.001) reduced the PE-induced contraction with cumulative concentrations of CaCl_2_ (0.1 a 1.0 mM): 123 ± 5% control *versus* 89 ± 7% Sn–I (1.0 mM CaCl_2_) (Fig. 6A and B). However, Ox–Sn–I did not decrease the PE-induced contraction in the free-calcium medium. No changes were found in EC_50_ for any bioactive molecules.

To ascertain whether Sn–I reduced the VGCC-mediated Ca^2+^ influx, vascular smooth muscle cells were stimulated with KCl (50 mM), and intracellular calcium levels were determined using fluorescence microscopy. In A7r5 cells preincubated with Sn–I and Ox–Sn–I (10^−5^ M), both compounds blunted the increased relative fluorescence (Fluo-4 AM) induced by KCl, but the effect of Sn–I was significantly higher than Ox–Sn–I (Fig. 6C and D).

The question addressed by the present study was whether a structural modification, involving the synthesis of oximes, could enhance the vascular response of plant bioactive molecules, exemplified by Sn–I. This study, for the first time, showed the Sn–I metabolite and its oxime cause endothelial vascular relaxation, and reduction of contractile response by blocking cytosolic calcium influx through the voltage-gated Ca^2+^ channel (VGCC) in the vascular smooth muscle cells.

Results showed that both compounds (Sn–I and its oxime Ox–Sn–I) produced vascular relaxation in intact aorta, in a dose-dependent manner. In addition, the absence of endothelium in aortic rings decreased relaxation at the submaximal dose, but it did not at the maximum dose. In the vascular endothelium, nitric oxide synthase (eNOS) is responsible for the generation of endothelial nitric oxide (NO), a main relaxing factor.^11^

The inhibition of eNOS with l-NAME confirmed that NO is involved in vascular relaxation induced by both Sn–I and Ox–Sn–I, respectively.

It is known that the oximes present R_2_C=NOH group that is metabolized by hemoproteins, hemoglobin and catalase, generating NO and vasodilatation,^12^ which is useful for treatment of hypertension.^13^ However, the NO generation using a fluorescence probe showed a small increase of endothelial NO in response to Sn–I and Ox–Sn–I in aortic rings compared to that induced by ACh. Since oximes generate NO,^14^ vascular endothelial relaxation by Ox–Sn–I should have been higher than Sn–I. One explanation would be that oxime did not produce NO efficiently, as observed in comparison to hydroxylamines, which are more potent vasoactive relaxant substances in rat aorta.^15^

The preincubation of rat aorta with Sn–I significantly reduced the contractile response to PE compared with Ox–Sn–I. Also, the sensitivity to PE significantly decreased in the presence of Sn–I, but not with Ox–Sn–I. It is known that PE is an α_1_ adrenergic agonist that causes vascular contraction through G protein-coupled receptor (GPCR) activation, mainly in vascular smooth muscle cells.^16^ In a free-calcium medium, the pre-incubation with Sn–I significantly decreased the adrenergic contraction to PE, suggesting that Sn–I blocked the release of calcium from the sarcoplasmic reticulum, and thus the stimulus to increase the influx of calcium, opening the store-operated Ca^2+^ channels (SOCC), and voltage-gated Ca^2+^ channels (VGCC).^17,18^ These findings are consistent with a cumulative concentration of extracellular calcium added to the medium, Sn–I significantly reduced the PE-induced contraction.

To ascertain whether Sn–I reduced the VGCC-mediated Ca^2+^ influx, vascular smooth muscle cells were stimulated with KCl, and intracellular calcium levels were determined using fluorescence microscopy. In A7r5 cells preincubated with Sn–I and Ox–Sn–I, both compounds blunted the increased relative fluorescence (Fluo-4 AM) induced by KCl, but the effect of Sn–I was significantly higher than Ox–Sn–I. KCl causes contraction in vascular smooth muscle cells through membrane depolarization, involving the activation of voltage-gated Ca^2+^ channel (VGCC), leading to the calcium influx.^19^

## Conclusion

**Fig. 7:**
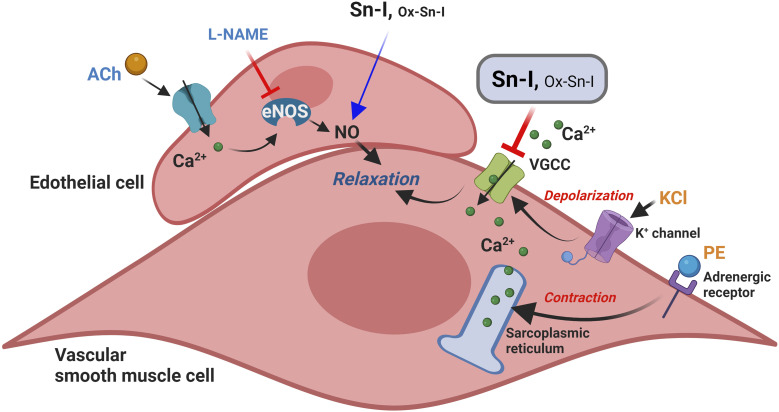
Putative model of relaxation and reduction of contraction of the Sn–I metabolite in rat blood vessels. The Sn–I metabolite decreases the contractile response to KCl or PE mainly by blocking Ca^2+^ entry through VGCC channels. KCl produces membrane depolarization, causing the entry of extracellular Ca^2+^ through VGCC channels. Furthermore, PE can selectively stimulate the alpha-adrenergic receptor on vascular smooth cells, leading to the release of Ca^2+^ from intracellular stores and an increase in Ca^2+^ influx through the VGCC.

Here, we showed that a derivate of 2,3-dihydrobenzofurane (also known as coumaran) from *S. nutans* (Sn–I) reduces the contractile response mainly by blocking Ca^2+^ entry through VGCC channels in vascular smooth cells.^20^ This effect also causes relaxation in rat aorta mainly mediated by the reduction of intracellular Ca^2+^, rather than an increase of NO generation in endothelial vascular cells (Fig. 7). We expected that chemical modification of Sn–I would produce an Ox–Sn–I oxime displaying a greater vascular relaxation effect and a reduction in the contractile response in rat aorta. However, this did not occur.

In summary, although both bioactive molecules cause relaxation in rat aorta mainly through blocking Ca^2+^ entry through VGCC channels in vascular smooth cells, the oxime effect was significantly lower than the metabolite. More chemical work is needed to find new bioactive molecules that enhance the vasodilatory effect of medicinal plant metabolites and provide new molecules for the treatment of hypertension.

## Experimental

### Chemicals

Hydroxylamine hydrochloride, pyridine, magnesium sulphate, l-phenylephrine hydrochloride (PE), acetylcholine chloride (ACh), N^G^-nitro-l-arginine methyl ester (l-NAME) were bought from Sigma-Aldrich (St Luis, MO, USA). The metabolite and oxime were dissolved in DMSO (0.1% final concentration).

### Isolation of natural products from *S. nutans*

The natural product 5-acetyl-6-hydroxy-2-isopropenyl-2,3-dihydrobenzofurane (Sn–I) was isolated from *S. nutans* according to a previous protocol described elsewhere.^4^ Briefly, the hydroalcoholic extract was resuspended in distilled water and extracted successively with *n*-hexane, dichloromethane (DCM) and ethyl acetate (EtOAc). Sn–I was isolated from dichloromethane subfraction. The organic solutions were concentrated on a rotary evaporator. The structural elucidation was carried out using spectroscopic data.

### Synthesis of oxime

Synthesis of oxime (Ox–Sn–I) was performed as previously described with a few modifications.^21^ To a solution of the keto-ester (500 mg, 2.5 mmol, 1.0) and hydroxylamine (180 mg, 2.5 mmol, 1.0) ethanol (10 mL) was added pyridine (1.6 mL × mmol) in 1 portion. The reaction mixture was heated at 65 °C for 24 h and then, concentrated on a rotary evaporator. The residue was partitioned between dichloromethane (DCM; 50 mL) and water (10 mL). The organic layer was sequentially washed with HCl (0.5 N) and water (10 mL), and then dried over anhydrous Na_2_SO_4_.

The purity of the isolated secondary metabolite and its oxime was monitored by TLC, comparing with laboratory reference samples, spectroscopic techniques and additionally its melting point was determined, which were compared with those described in the literature.

### Animals

Male Sprague Dawley rats (6–8 weeks old; *n* = 12) weighing between 170 g and 200 g were used in this study. All animal procedures were performed in accordance with the Guidelines for Care and Use of Laboratory Animals of Universidad de Antofagasta and experiments were approved by the Animal Ethics Committee of Universidad de Antofagasta (CEIC #275/2020). The animals were housed in plastic cages at room temperature of 22–25 °C and humidity of 45–51% and had full access to tap water and food (*ad libitum*).

### Isolation of rat aorta and vascular reactivity assays

This procedure was performed based on the method previously described.^4^ Animals were euthanized by cervical dislocation. The aortic rings were placed in organ bath with Krebs–Ringer bicarbonate (KRB) solution (in mM); 4.2 KCl, 1.19 KH_2_PO_4_, 120 NaCl, 25 NaHCO_3_, 1.2 MgSO_4_, 1.3 CaCl_2_, and 5 d-glucose, pH 7.4, 37 °C, 95% O_2_ and 5% CO_2_. After the equilibration period for 30 min, the aortic rings were stabilized by 3 successive near-maximum contractions with KCl (60 mM) for 10 min. The integrity of the vascular endothelium was assessed using 10^−5^ M acetylcholine (ACh). The passive tension on aorta was 1.0 g, which was determined to be the resting tension for obtaining maximum active tension induced by 60 mM KCl.

To evaluate the contractile response to phenylephrine (PE, 10^−10^ to 10^−5^ M) or KCl (10 to 60 mM), the tissue was pre-incubated in KRB for 20 min prior to contraction. In an alternative experimental approach, the relaxation capacity of the extract or isolated metabolite was examined. In this scenario, the tissue was pre-contracted with 10^−6^ M PE, and escalating concentrations of bioactive molecules were introduced to the organ bath during the vascular plateau response.

### Intracellular Ca^2+^ and NO measurements

To confirm the role of the intracellular Ca^2+^ and NO in the vascular response, we determined intracellular Ca^2+^ and NO levels in the vascular smooth muscle cell line A7r5 (ATCC CRL-1444) and in aortic ring slices (<1 mm), respectively. Intracellular Ca^2+^ determinations^22^ and for NO measurements^23^ were performed as previously described. Cells were cultured in coverslips and incubated with 10 μM Fluo-4 AM or preincubated aortic rings with 5 μM 4-amino-5-methylamino-2′,7′-difluorofluorescein (DAF-FM) diacetate (Thermo Fisher Scientific) in KRB for 30 min at 37 °C. Cells were placed on a 1 mL chamber in a Carl Zeiss LSM-5 Pascal 5 Axiovert 200 microscope, excited with 488 nm (500 nm for DAF-FM DA) and the emitted fluorescence monitored at 527 nm (515 nm for DAF-FM DA). Cells or tissues were pretreated with both compounds, Sn–I or Ox–Sn–I (10^−5^ M), or vehicle for 30 min. Images of 4–5 different experiments were collected every 1 s and analyzed frame-by-frame with ImageJ software (NIH).^24^ Intracellular Ca^2+^ levels are expressed as relative fluorescence, Δ*F*/*F*_0_, where Δ*F* represents the difference between the experimental value *F* and the basal fluorescence value *F*_0_.

### Statistical analysis

The results obtained from the experiments are expressed as mean ± standard error of the mean (SEM). Statistical analysis of the data was performed using analysis of variance (two-way ANOVA) followed by Bonferroni *post hoc* test. In addition, the determination of the sensitivity (EC_50_ or IC_50_) was performed using nonlinear regression (sigmoidal) *via* Graph Pad Prism software, version 5.0 (GraphPad Software, Inc., La Jolla, CA, USA). Statistical significance is set at *p* < 0.05.

## Author contributions

Conceptualization: Adrián Paredes, Javier Palacios, and Fredi Cifuentes; formal analysis and investigation: Fredi Cifuentes, Daniel Asunción-Alvarez, Diego Aravena, Adrián Paredes, Javier Palacios; writing—original draft preparation: Daniel Asunción-Alvarez, Javier Palacios, and Adrián Paredes; writing—review and editing: Marcelo Catalán, Javier Palacios, Claudio Parra, Mario Chiong. All authors have read and agreed to the published version of the manuscript.

## Conflicts of interest

No conflicts of interest, financial or otherwise, are declared by the authors.

## Supplementary Material

RA-014-D4RA01058B-s001

## References

[cit1] Al Disi S. S., Anwar M. A., Eid A. H. (2016). Anti-hypertensive Herbs and their Mechanisms of Action: Part I. Front. Pharmacol.

[cit2] Trevizan J., Soto E., Parra F., Bustos L., Parra C. (2020). Antioxidant activity of nine medicinal plants with commercial potential. Idesia.

[cit3] Cifuentes F., Paredes A., Palacios J., Muñoz F., Carvajal L., Nwokocha C. R. (2016). *et al.*, Hypotensive and antihypertensive effects of a hydroalcoholic extract from Senecio nutans Sch. Bip. (Compositae) in mice: Chronotropic and negative inotropic effect, a nifedipine-like action. J. Ethnopharmacol..

[cit4] Paredes A., Palacios J., Quispe C., Nwokocha C. R., Morales G., Kuzmicic J., Cifuentes F. (2016). Hydroalcoholic extract and pure compounds from Senecio nutans Sch. Bip (Compositae) induce vasodilation in rat aorta through endothelium-dependent and independent mechanisms. J. Ethnopharmacol..

[cit5] Cifuentes F., Palacios J., Kuzmicic J., Carvajal L., Muñoz F., Quispe C. (2018). *et al.*, Vasodilator and hypotensive effects of pure compounds and hydroalcoholic extract of Xenophyllum poposum (Phil) V.A Funk (Compositae) on rats. Phytomedicine.

[cit6] Giberti G. C. (1983). Herbal folk medicine in northwestern Argentina: Compositae. J. Ethnopharmacol..

[cit7] Palacios J., Paredes A., Cifuentes F., Catalán M. A., García-Villalón A. L., Borquez J. (2023). *et al.*, A hydroalcoholic extract of Senecio nutans SCh. Bip (Asteraceae); its effects on cardiac function and chemical characterization. J. Ethnopharmacol..

[cit8] Palacios J., Paredes A., Catalán M. A., Nwokocha C. R., Cifuentes F. (2022). Novel Oxime Synthesized from a Natural Product of Senecio nutans SCh. Bip. (Asteraceae) Enhances Vascular Relaxation in Rats by an Endothelium-Independent Mechanism. Molecules.

[cit9] Sahyoun T., Arrault A., Schneider R. (2019). Amidoximes and Oximes: Synthesis, Structure, and Their Key Role as NO Donors. Molecules.

[cit10] Leloup A. J. A., Van Hove C. E., De Moudt S., De Keulenaer G. W., Fransen P. (2020). Ex vivo aortic stiffness in mice with different eNOS activity. Am. J. Physiol.: Heart Circ. Physiol..

[cit11] Moncada S., Palmer R. M., Higgs E. A. (1991). Nitric oxide: physiology, pathophysiology, and pharmacology. Pharmacol. Rev..

[cit12] Dantas B. P., Ribeiro T. P., Assis V. L., Furtado F. F., Assis K. S., Alves J. S. (2014). *et al.*, Vasorelaxation induced by a new naphthoquinone-oxime is mediated by NO-sGC-cGMP pathway. Molecules.

[cit13] Ghabbour H. A., El-Bendary E. R., El-Ashmawy M. B., El-Kerdawy M. M. (2014). Synthesis, Docking Study and beta-Adrenoceptor Activity of Some New Oxime Ether Derivatives. Molecules.

[cit14] Sahyoun T., Arrault A., Schneider R. (2019). Amidoximes and oximes: Synthesis, structure, and their key role as No donors. Molecules.

[cit15] Thomas G., Ramwell P. W. (1989). Vascular relaxation mediated by hydroxylamines and oximes: Their conversion to nitrites and mechanism of endothelium dependent vascular relaxation. Biochem. Biophys. Res. Commun..

[cit16] Fan L. L., Ren S., Zhou H., Wang Y., Xu P. X., He J. Q. (2009). *et al.*, α1D-Adrenergic receptor insensitivity is associated with alterations in its expression and distribution in cultured vascular myocytes. Acta Pharmacol. Sin..

[cit17] Liu Z., Khalil R. A. (2018). Evolving mechanisms of vascular smooth muscle contraction highlight key targets in vascular disease. Biochem. Pharmacol..

[cit18] Touyz R. M., Alves-Lopes R., Rios F. J., Camargo L. L., Anagnostopoulou A., Arner A. (2018). *et al.*, Vascular smooth muscle contraction in hypertension. Cardiovasc. Res..

[cit19] Ratz P. H., Berg K. M., Urban N. H., Miner A. S. (2005). Regulation of smooth muscle calcium sensitivity: KCl as a calcium-sensitizing stimulus. Am. J. Physiol.: Cell Physiol..

[cit20] Jackson W. F. (2017). Potassium Channels in Regulation of Vascular Smooth Muscle Contraction and Growth. Adv. Pharmacol..

[cit21] Hart D. J., Magomedov N. A. (2001). Synthesis of ent-alantrypinone. J. Am. Chem. Soc..

[cit22] Cifuentes F., Palacios J., R Nwokocha C., Bórquez J., Simirgiotis M. J., Norambuena I. (2019). *et al.*, Polyphenolic Composition and Hypotensive Effects of Parastrephia quadrangularis (Meyen) Cabrera in Rat. Antioxidants.

[cit23] Berra-Romani R., Avelino-Cruz J. E., Raqeeb A., Della Corte A., Cinelli M., Montagnani S. (2013). *et al.*, Ca2+-dependent nitric oxide release in the injured endothelium of excised rat aorta: A promising mechanism applying in vascular prosthetic devices in aging patients. BMC Surg..

[cit24] Schneider C. A., Rasband W. S., Eliceiri K. W. (2012). NIH Image to ImageJ: 25 years of image analysis. Nat. Methods.

