# Supplementary material for: A new oxime synthesized from Senecio nutans SCh. Bip (chachacoma) reduces calcium influx in the vascular contractile response in rat aorta
Source: RSC Adv. 2024 Mar 25;14(14):9933–42. doi: 10.1039/d4ra01058b (PMC10962255; doi:10.1039/d4ra01058b)
Supplement: RA-014-D4RA01058B-s001 [file RA-014-D4RA01058B-s001.pdf]

Electronic Supplementary Material (ESI) for RSC Advances.
This journal is © The Royal Society of Chemistry 2024

Supplementary information

Reduction of Calcium Influx in the Vascular Contractile Response in Rat Aorta by a New Oxime Synthesized from *Senecio nutans* SCh. Bip (Chachacoma)

Javier Palacios [a*], Daniel Asunción-Alvarez [a], Diego Aravena [a], Mario Chiong [b], Marcelo A. Catalán [c], Claudio Parra [d], Fredi Cifuentes [e], Adrián Paredes [f*]

[a.] *Laboratorio de Bioquímica Aplicada, Facultad de Ciencias de la Salud, Universidad Arturo Prat, Iquique 1110939, Chile. clpalaci@unap.cl (J.P.); holbertasuncion.pharm@gmail.com (D.A.A.); diego.andresx21@gmail.com (D.A.)*
[b.] *Universidad de Chile, Advanced Center for Chronic Diseases (ACCDiS), Facultad de Ciencias Químicas y Farmacéuticas, Santiago, Chile. mchiong@ciq.uchile.cl (M.C.)*
[c.] *Instituto de Fisiología, Facultad de Medicina, Universidad Austral de Chile, Valdivia 5090000, Chile. marcelo.catalan@uach.cl (M.A.C.)*
[d.] *Departamento de Química Orgánica, Facultad de Ciencias Químicas, Universidad de Concepción, Edmundo Larenas 129, Concepción 4070371, Chile. cparra@udec.cl (C.P.)*
[e.] *Laboratorio de Fisiología Experimental (EPhyL), Instituto Antofagasta (IA), Universidad de Antofagasta, Antofagasta 1271155, Chile. fredi.cifuentes.jorquera@gmail.com (F.C.)*
[f.] *Departamento de Química, Facultad de Ciencias Básicas, Universidad de Antofagasta, Antofagasta 1271155, Chile. adrian.paredes@uantof.cl (A.P.)*

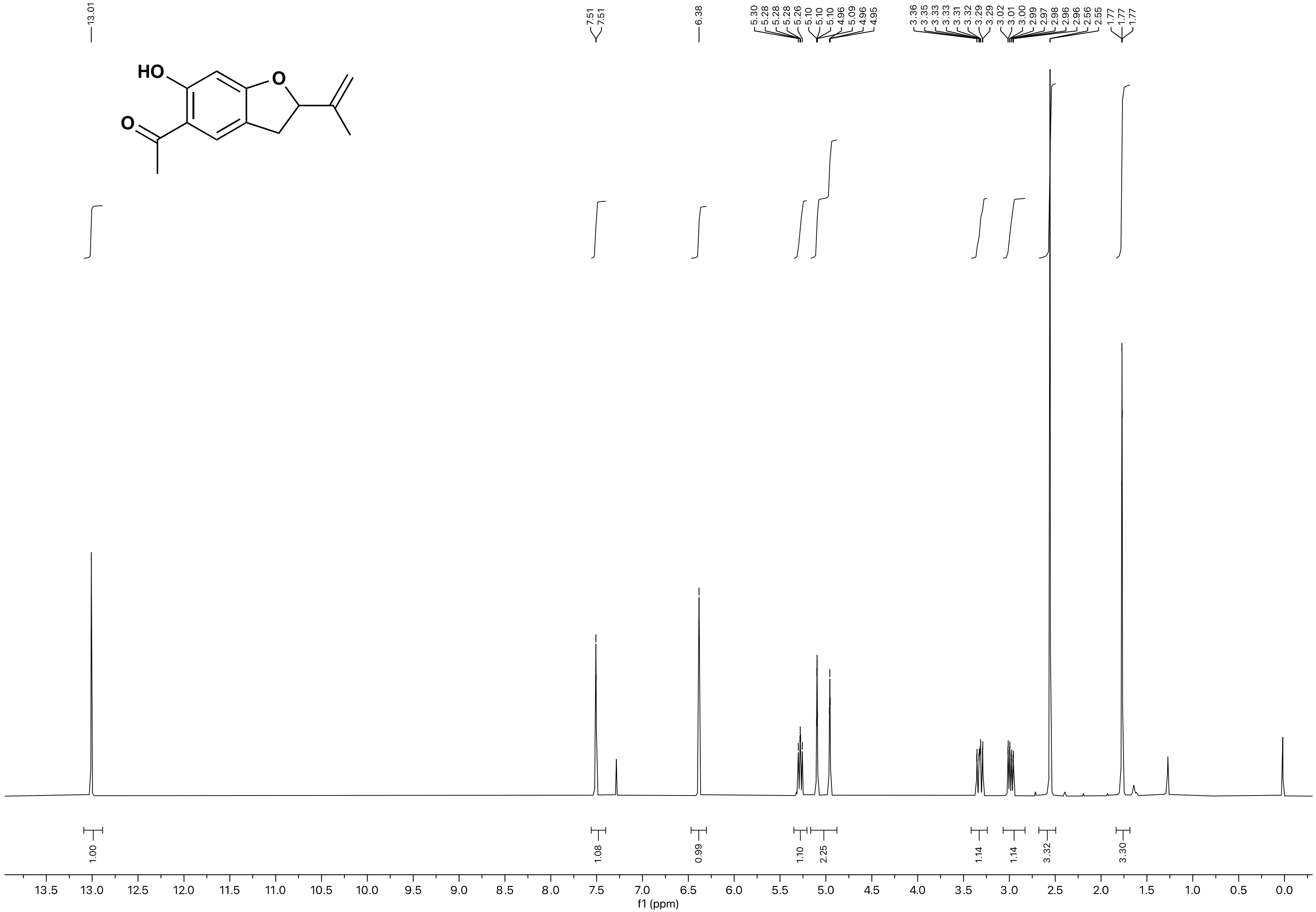

Supplementary figure 1.- ^{1}H NMR spectrum of 6-hydroxy-2-isopropenyl-5-acetyl-2,3-dihydrobenzofuran

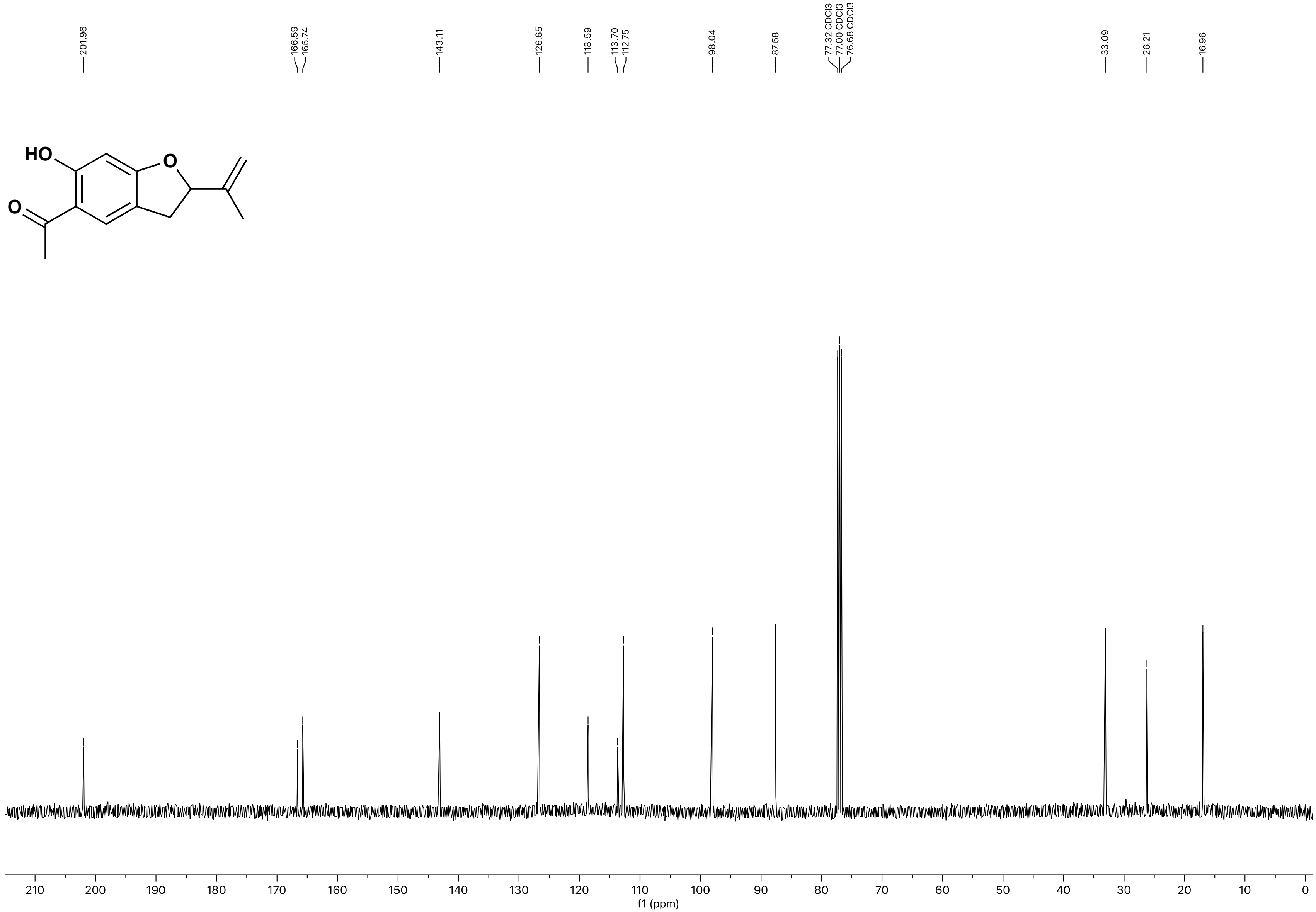

Supplementary figure 2.- ^{13}C NMR spectrum of 6-hydroxy-2-isopropenyl-5-acetyl-2,3-dihydrobenzofuran

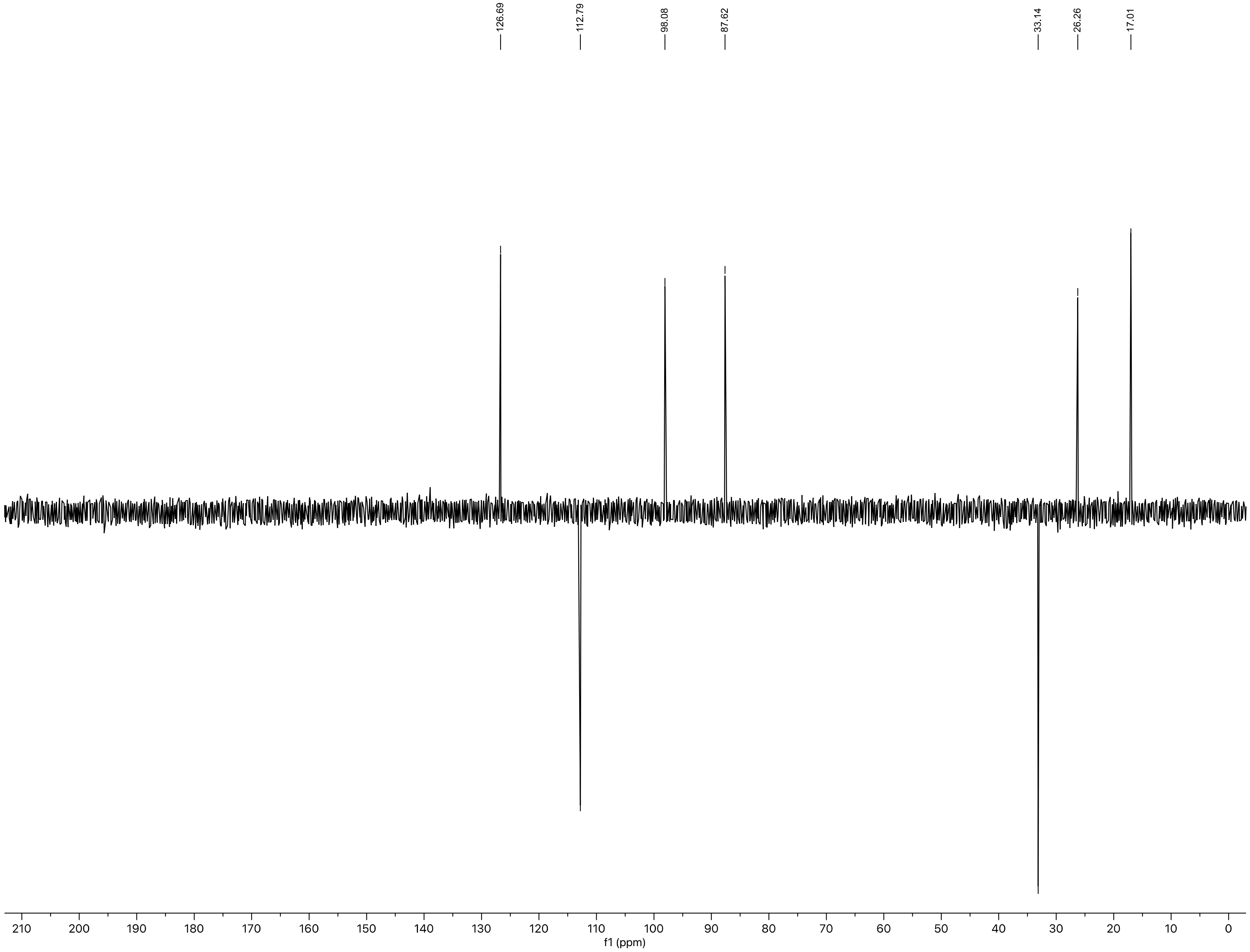

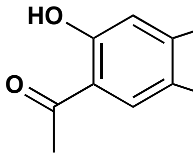

Supplementary figure 3.- DEPT135 spectrum of 6-hydroxy-2-isopropenyl-5-acetyl-2,3-dihydrobenzofuran

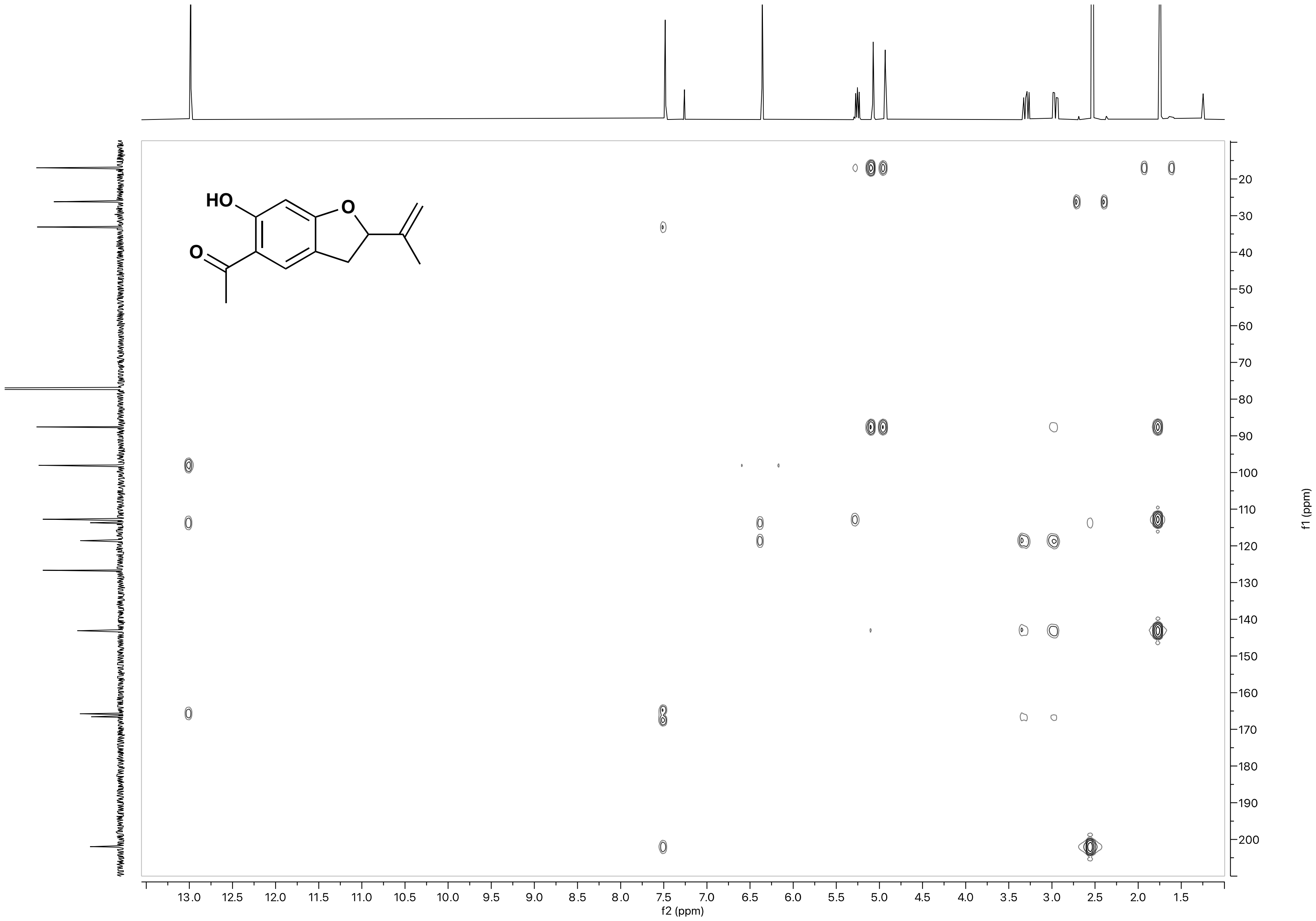

Supplementary figure 4.- HMBC spectrum of 6-hydroxy-2-isopropenyl-5-acetyl-2,3-dihydrobenzofuran

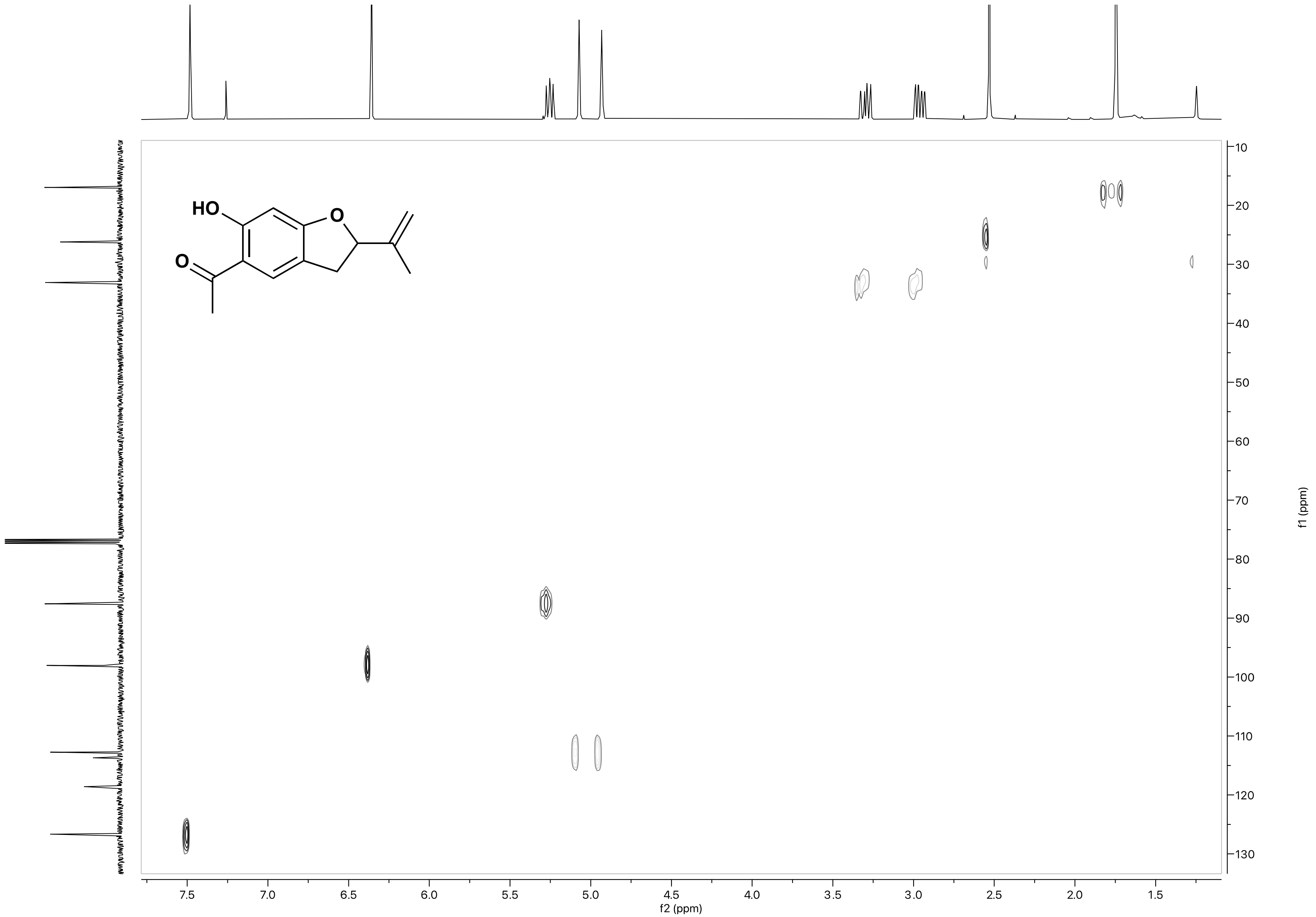

Supplementary figure 5.- HMQC spectrum of 6-hydroxy-2-isopropenyl-5-acetyl-2,3-dihydrobenzofuran

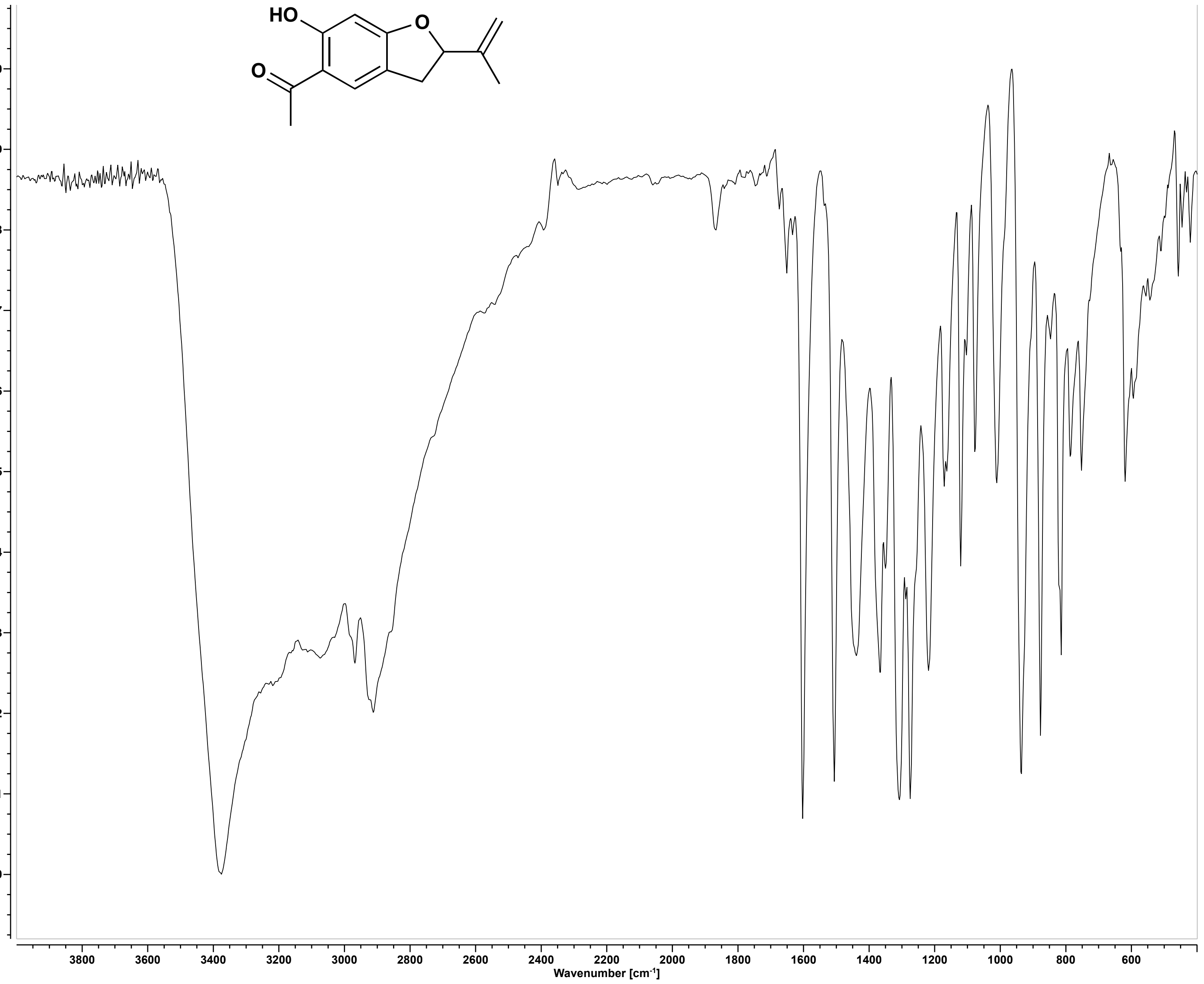

Supplementary figure 6.- FT-IR spectrum of 6-hydroxy-2-isopropenyl-5-acetyl-2,3-dihydrobenzofuran

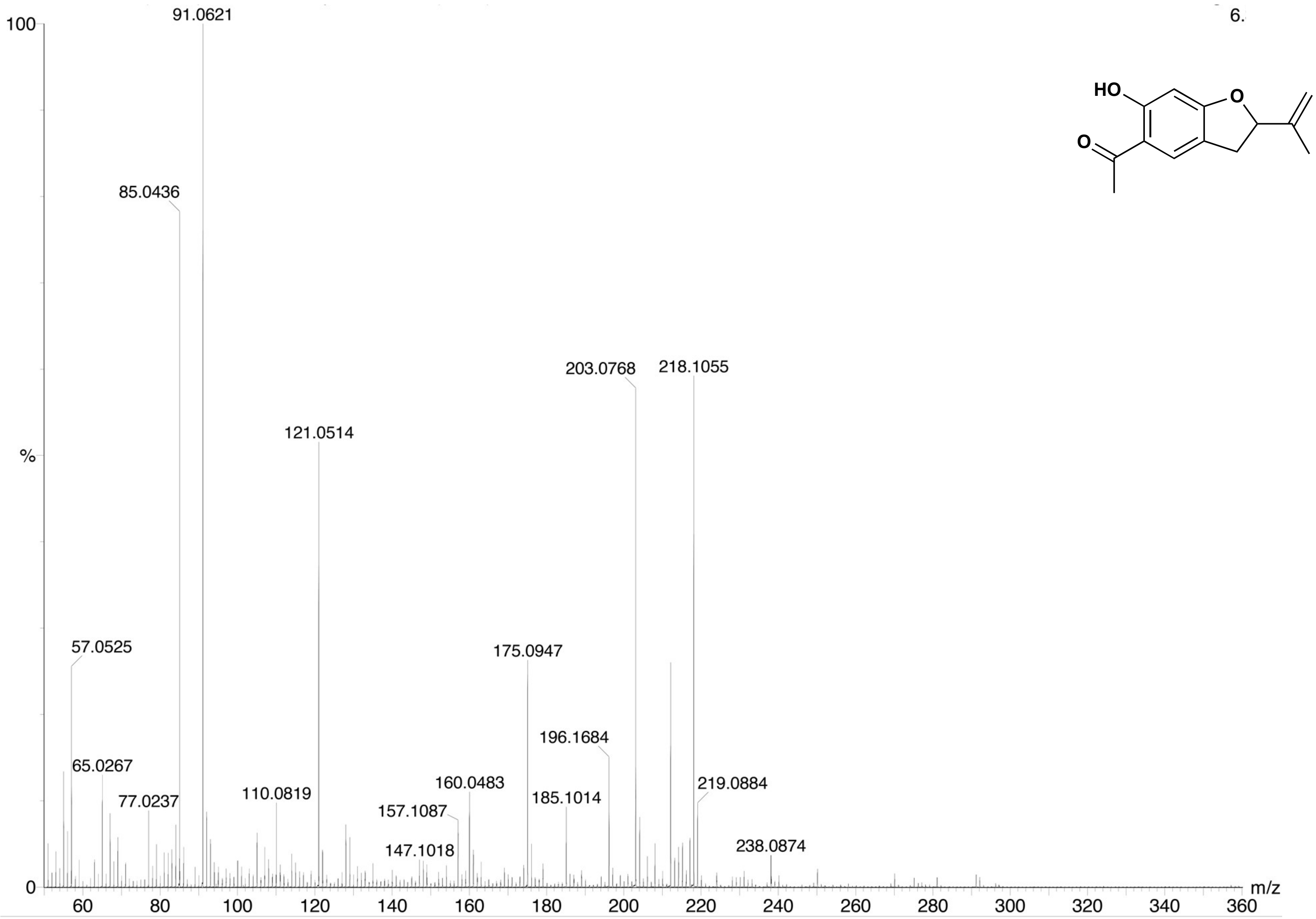

Supplementary figure 7.- HR EI-MS spectrum of 6-hydroxy-2-isopropenyl-5-acetyl-2,3-dihydrobenzofuran

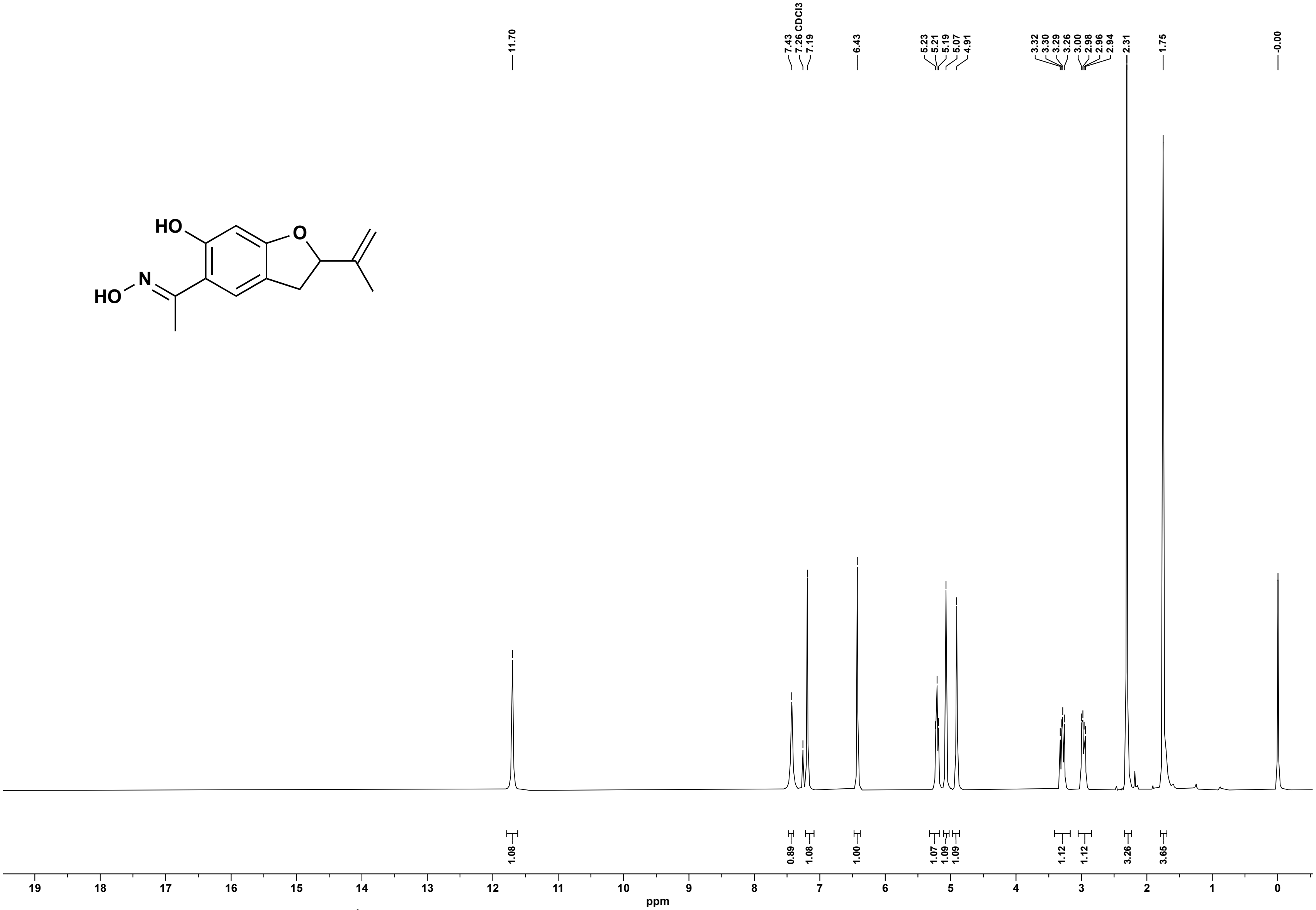

Supplementary figure 8.- ^{1}H NMR spectrum of 6-hydroxy-2-isopropenyl-5-acetyloxime-2,3-dihydrobenzofuran

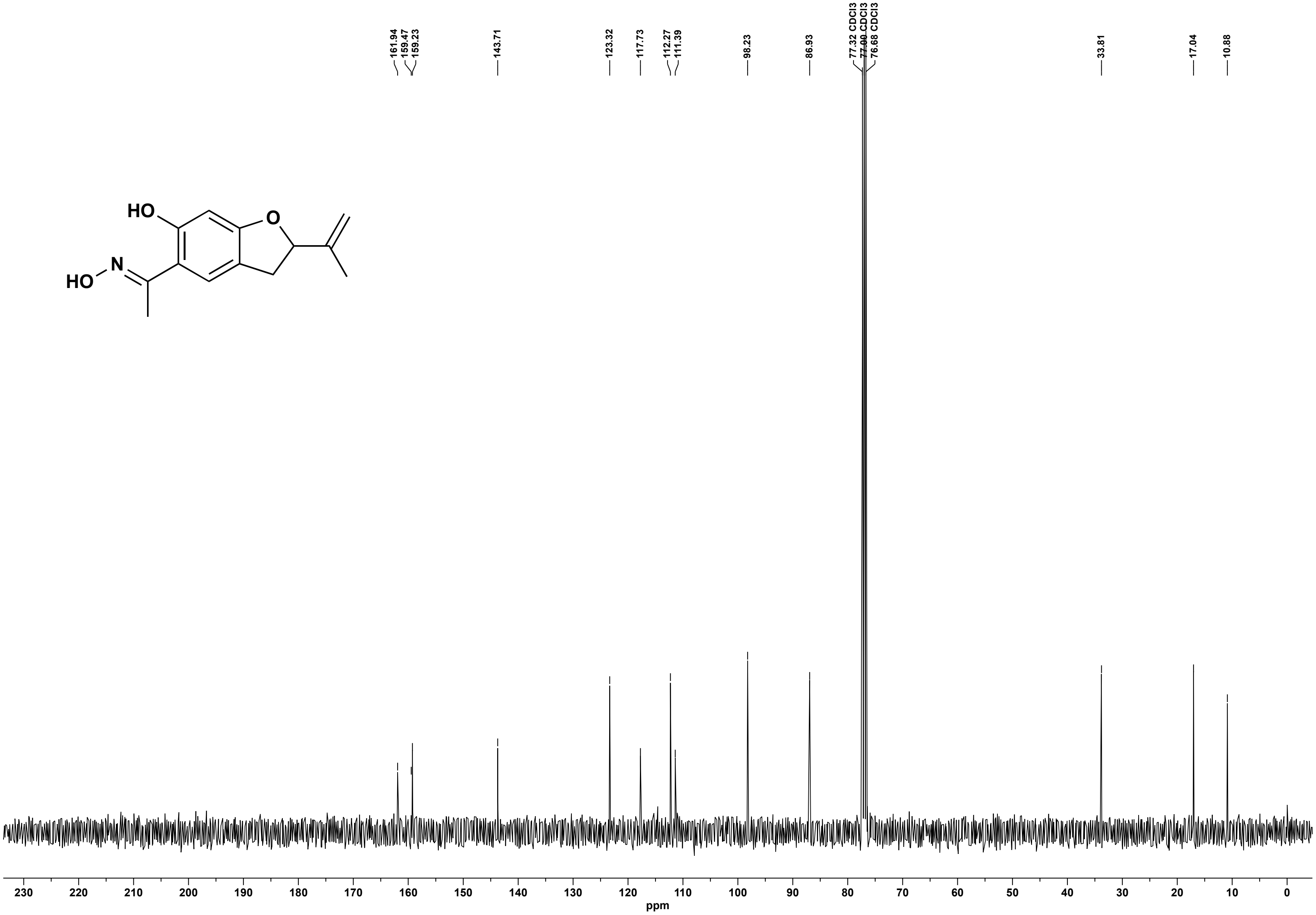

Supplementary figure 9.- ^{13}C NMR spectrum of 6-hydroxy-2-isopropenyl-5-acetyloxime-2,3-dihydrobenzofuran

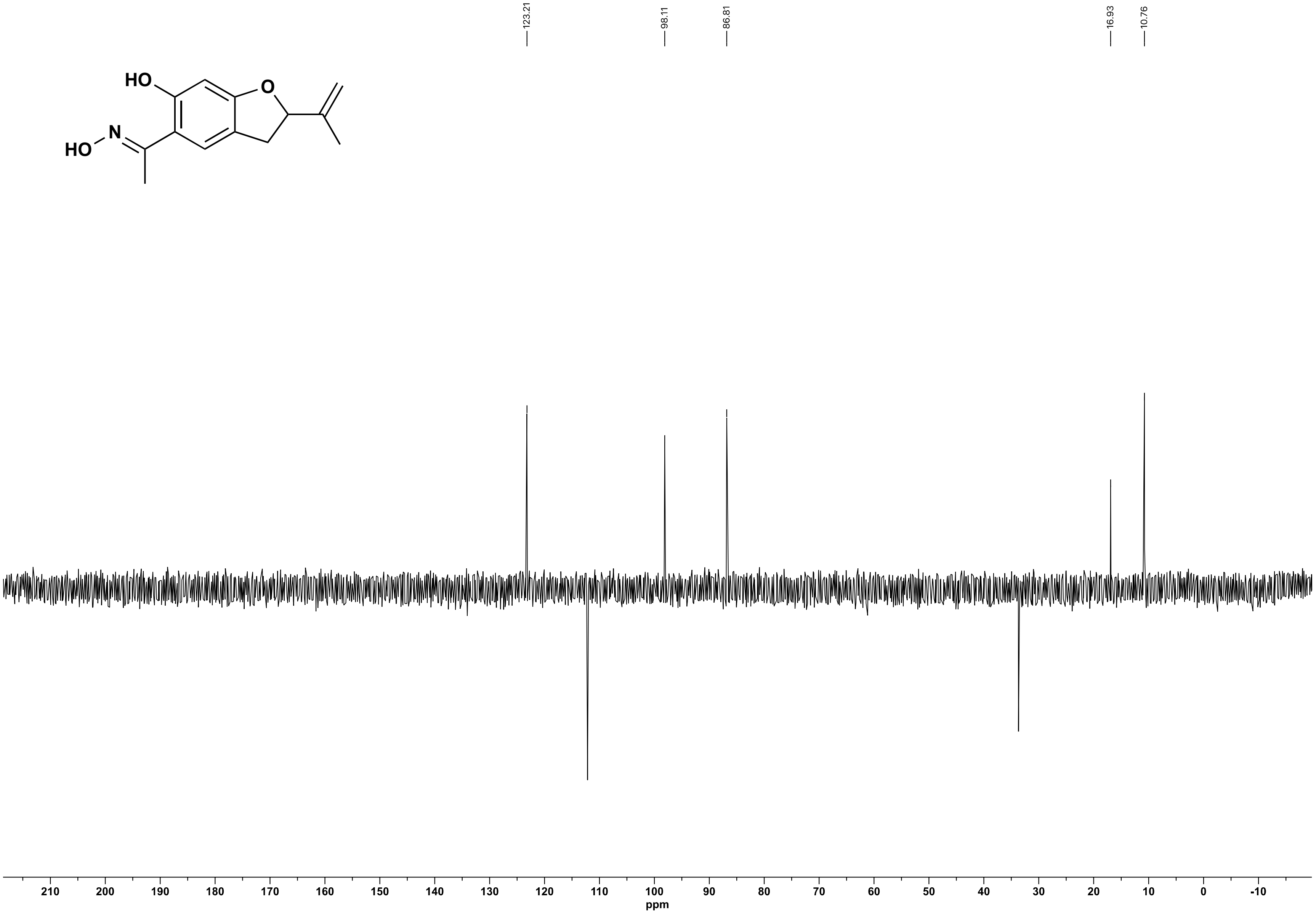

Supplementary figure 10.- DEPT135 spectrum of 6-hydroxy-2-isopropenyl-5-acetyloxime-2,3-dihydrobenzofuran

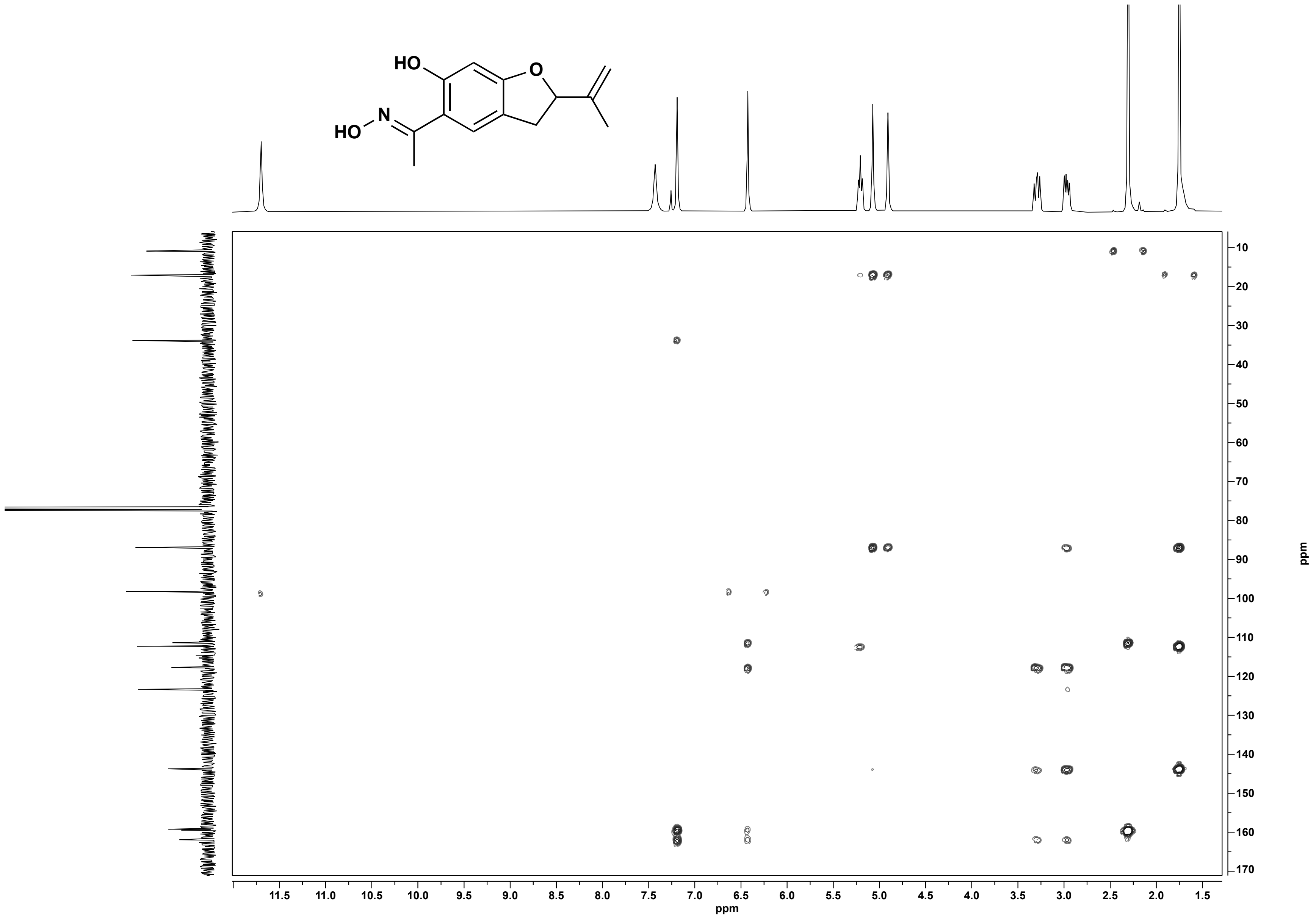

Supplementary figure 11.- HMBC spectrum of 6-hydroxy-2-isopropenyl-5-acetyloxime-2,3-dihydrobenzofuran

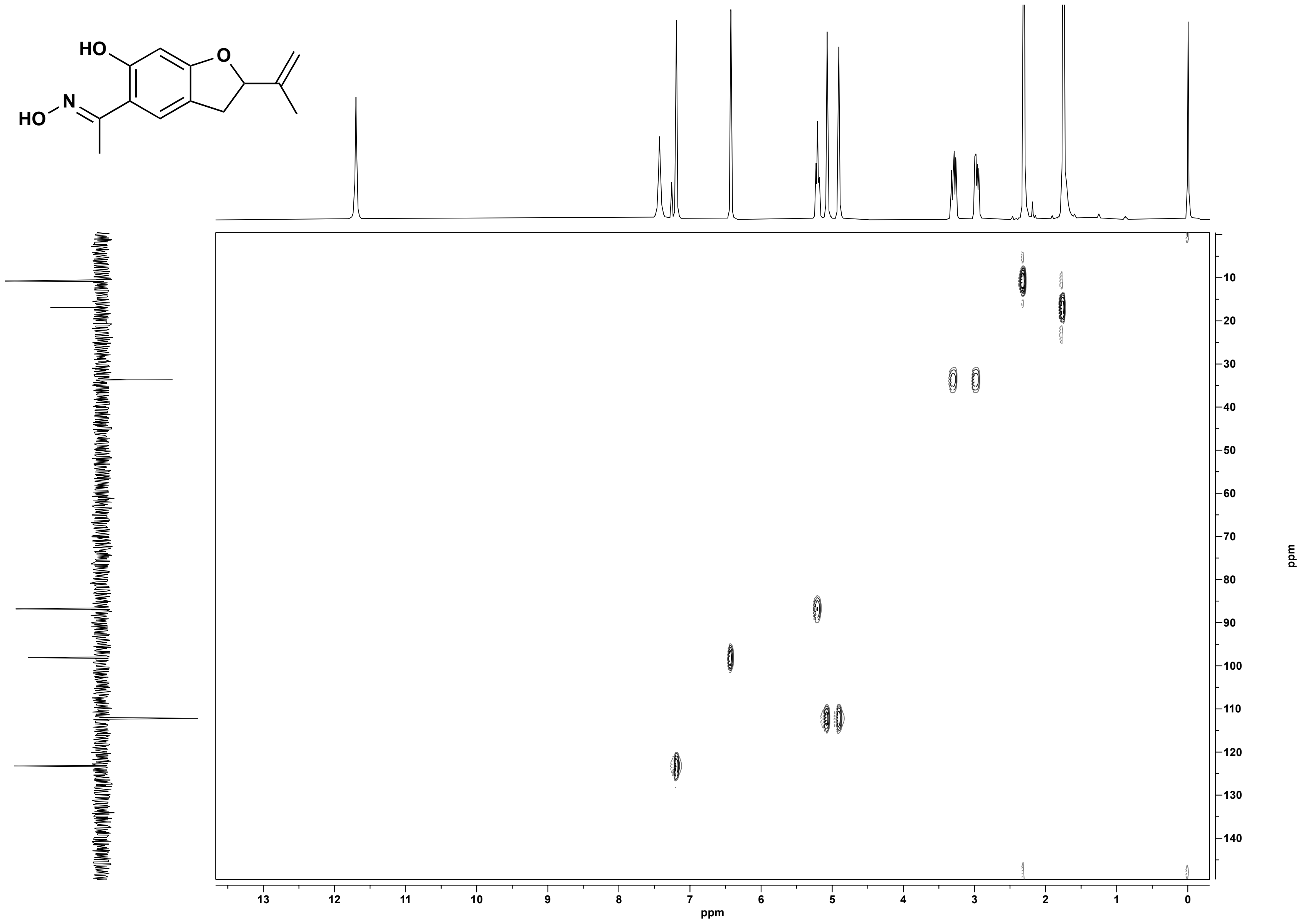

Supplementary figure 12.- HMQC spectrum of 6-hydroxy-2-isopropenyl-5-acetyloxime-2,3-dihydrobenzofuran

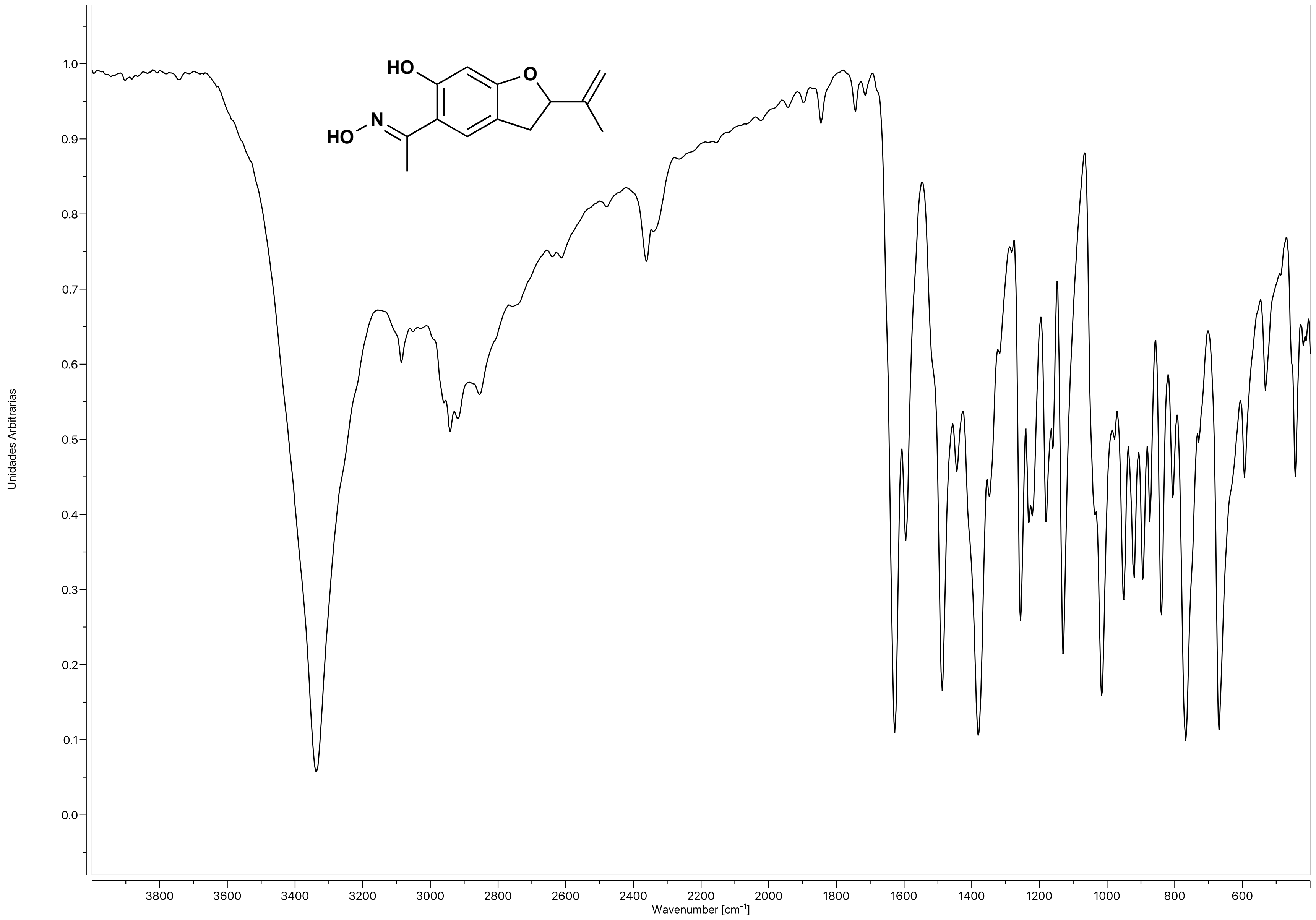

Supplementary figure 13.- FT-IR spectrum of 6-hydroxy-2-isopropenyl-5-acetyloxime-2,3-dihydrobenzofuran

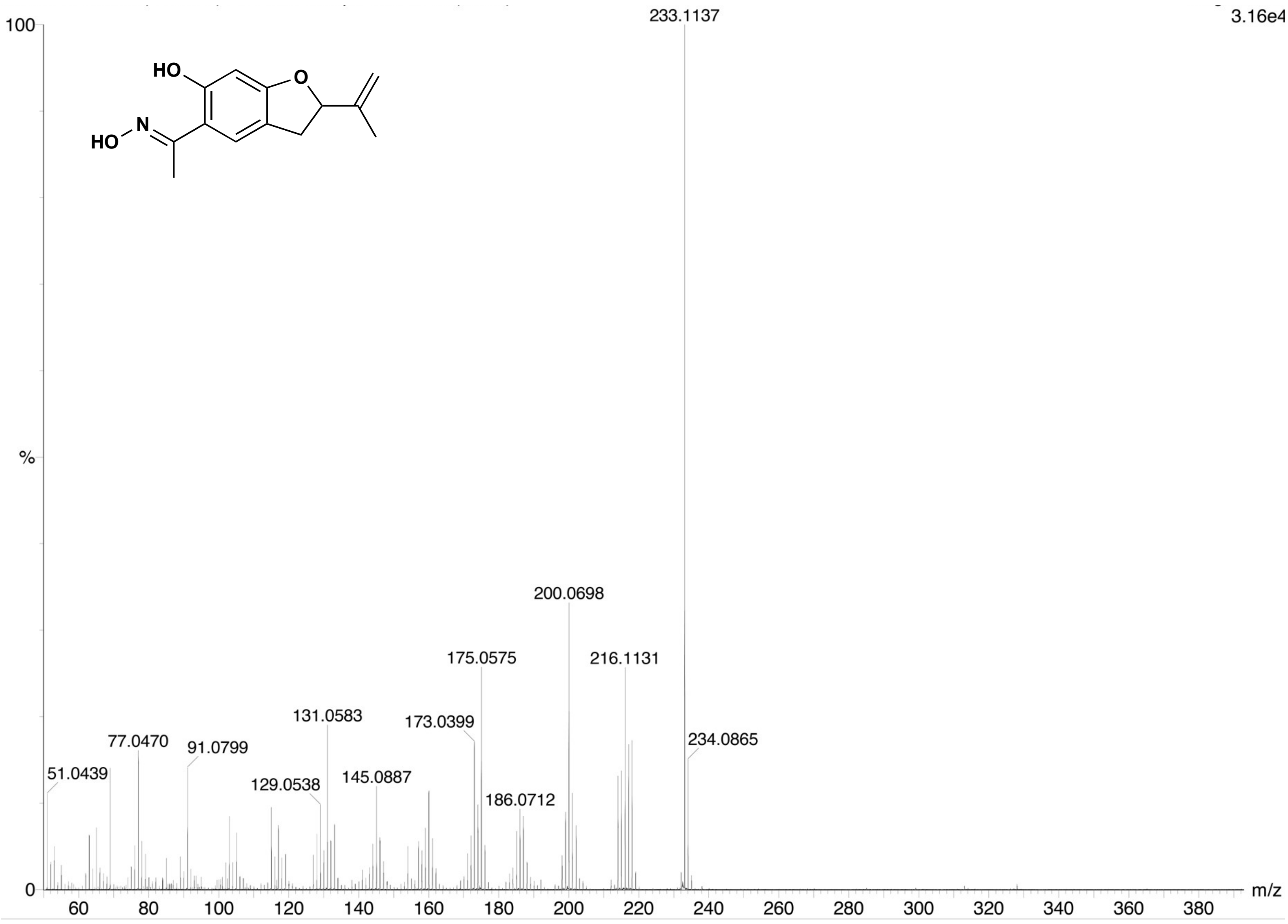

Supplementary figure 14.- HR EI-MS spectrum of 6-hydroxy-2-isopropenyl-5-acetyloxime-2,3-dihydrobenzofuran
